# Disrupted routines anticipate musical exploration

**DOI:** 10.1073/pnas.2306549121

**Published:** 2024-02-01

**Authors:** Khwan Kim, Noah Askin, James A. Evans

**Affiliations:** ^a^Area of Organizational Behaviour, INSEAD, Fontainebleau 77300, France; ^b^Department of Organization and Management, The Paul Merage School of Business, University of California–Irvine, Irvine, CA 92697; ^c^Department of Sociology, University of Chicago, Chicago, IL 60637; ^d^Knowledge Lab, University of Chicago, Chicago, IL 60637

**Keywords:** cultural analytics, cultural consumption, taste exploration, human mobility, social disruption

## Abstract

Understanding and predicting the emergence and evolution of cultural tastes manifested in consumption patterns is of central interest to social scientists, analysts of culture, and purveyors of content. Prior research suggests that taste preferences relate to personality traits, values, shifts in mood, and immigration destination. Understanding everyday patterns of listening and the function music plays in life has remained elusive, however, despite speculation that musical nostalgia may compensate for local disruption. Using more than one hundred million streams of four million songs by tens of thousands of international listeners from a global music service, we show that breaches in personal routine are systematically associated with personal musical exploration. As people visited new cities and countries, their preferences diversified, converging toward their travel destinations. As people experienced the very different disruptions associated with COVID-19 lockdowns, their preferences diversified further. Personal explorations did not tend to veer toward the global listening average, but away from it, toward distinctive regional musical content. Exposure to novel music explored during periods of routine disruption showed a persistent influence on listeners’ future consumption patterns. Across all of these settings, musical preference reflected rather than compensated for life’s surprises, leaving a lasting legacy on tastes. We explore the relationship between these findings and global patterns of behavior and cultural consumption.

## Significance

The emergence and evolution of cultural tastes is of central interest to cultural purveyors, producers, and social scientists. Prior research suggests that taste preferences relate to intrinsic, learned, and ephemeral personal qualities and contexts. Using more than one hundred million musical streams by tens of thousands of international listeners, we demonstrate that despite speculation that musical nostalgia compensates for life’s disruptions, musical exploration systematically reflects breaches in personal routine. As people visited new cities and countries, their preferences diversified, converging toward their destinations. As people experienced COVID-19 lockdowns, their preferences diversified further, veering away from the global average toward distinctive regional content. These musical explorations manifest a lasting influence on the user’s future cultural consumption.

Understanding and anticipating the emergence and evolution of cultural tastes and consumption patterns remains the holy grail for analysts of culture (1–3) and companies that deliver cultural content: Netflix has held contests to identify who could design a better recommendation algorithm for suggesting content to its users, TikTok has raced to the top of the mobile application world with “addictive” short video recommendations that better capture and align with users’ tastes, and digital streaming platforms (DSPs) in music compete on both quality of recommendation engine and size of available library. Artists, companies, and scholars alike seek to understand and influence users’ tastes—an endeavor made easier by the increase in digital trace data these services generate (4–7). Yet examining what shapes taste preferences remains a challenge, as they change over time and across contexts, and as the quantity of available culture explodes. Aside from the rise in mood- and context-based playlists for music (e.g., “Mood Booster,” “Rainy Day” or “Morning Commute” playlists on Spotify), a trend that aligns with evidence showing that musical preferences are associated with context and mood (8, 9), there is little evidence demonstrating how tastes are influenced by everyday shifts in routine and context. We propose and demonstrate that disruptions in routine and shifts in context vary positively with cultural consumption and listening habits.

Here, we build on work linking cultural taste preferences to consumers’ intrinsic characteristics (10–13) and external environment (14–16) by characterizing both travel–vacations, business trips, etc., and COVID-19-induced lockdowns as disruptions in consumers’ routines that influence consumption behaviors. In this context, we explore how taste and the functional roles of music (17–19) evolve, particularly within unfamiliar circumstances. Specifically, we scrutinize monthly patterns of spatial change—quantified by the geographical distances consumers travel—and their influence on individuals’ inclination to venture outside their habitual cultural consumption patterns. This measure is captured as the distance between an individual’s past taste and their new monthly listening behaviors. We further investigate the potential of COVID-19 lockdowns as catalysts for shifts in consumption patterns.

Our study delves into an empirical evaluation of Anne Swidler’s foundational Sociology of Culture theory regarding “unsettled times” and “unsettled lives” (20). Swidler posits that periods of upheaval—when social norms and relationships are in flux and culture’s prescriptive role weakens—can give rise to cultural innovation and openness to change. According to her theory, individuals adopt and deploy new cultural ideologies during such periods to navigate uncertainty. This notion aligns with psychological insights indicating that divergent or atypical stimuli “drive” curiosity and intrinsic motivations to explore in human adults, children, and nonhuman mammals (21–28). These stimuli, whether novel environments, altered socio-cultural landscapes, or atypical products themselves, possess an inherent reward value that makes exploration attractive and fulfilling. This, in turn, increases a positive evaluation of novelty and thereby increases the likelihood of consuming novel products (21, 29). Here, we consider how novel cultural exposures during “unsettled” periods act in the same way, reshaping aesthetic experiences and tastes in ways that persist. By examining both personal travel-induced disruptions (“unsettled lives”) and the collective dislocation caused by COVID-19 lockdowns (“unsettled times”), we offer a broad test of Swidler’s theory in the context of cultural consumption. Notably, animal research has found evidence for novelty seeking both in the presence of disruptive stimuli (“unsettled times”) and disorienting boredom (“unsettled lives”) (28). By analyzing these dynamics in a global comparative context, we deepen our understanding of the dynamic interplay between socio-cultural environments and human action during and after periods of disruption.

## The Construction of Taste Vectors for Preference Measurement

**Fig. 1.**
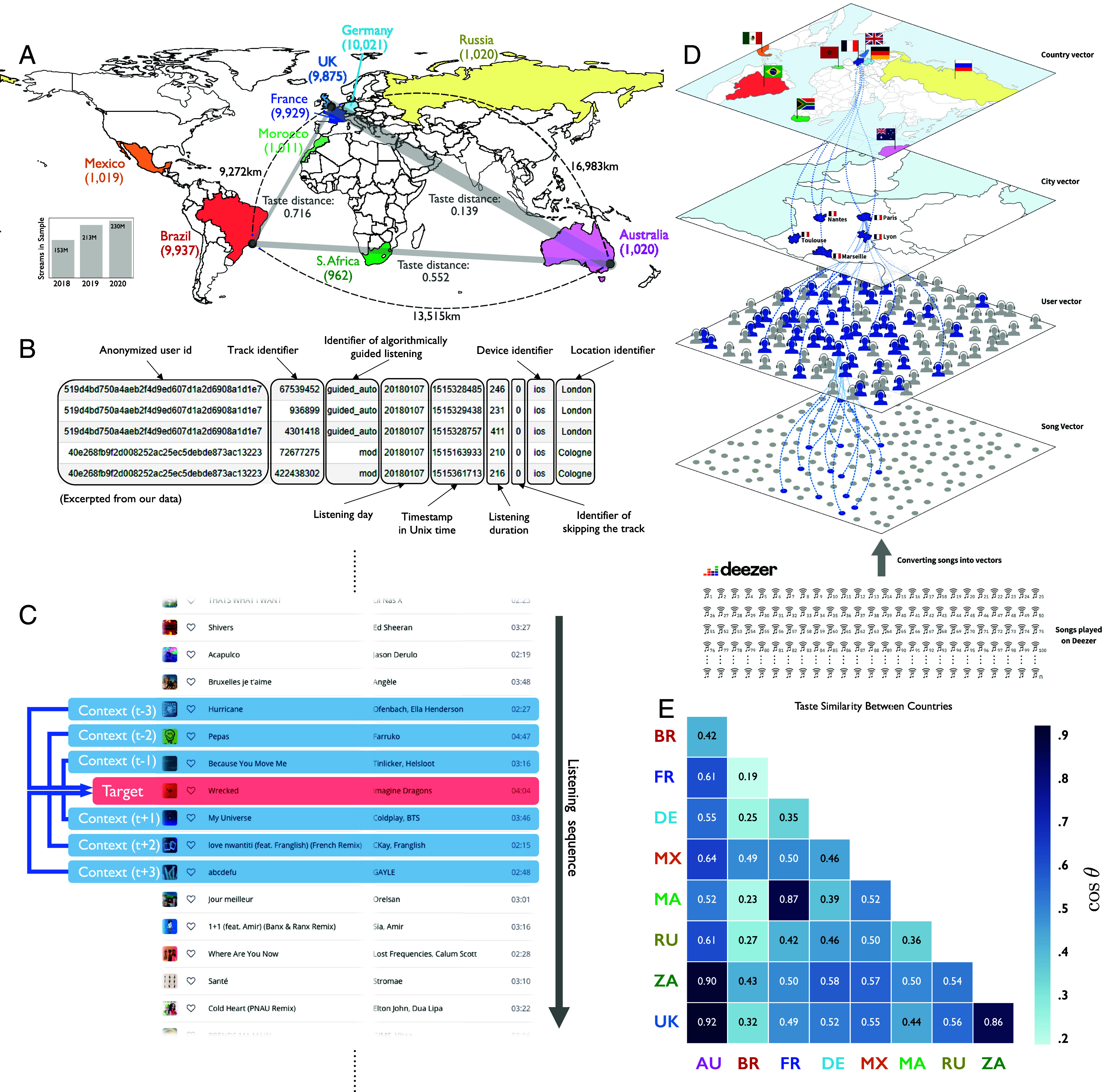
Data and measurement overview. (*A*) Countries and continents of users in sample, number of musical streams per year, and distinctions between geographical distance and musical/taste distances emergent from streaming songs-in-context. (*B*) Annotated sample of streaming data fields. (*C*) Arrangement of streamed songs in order to determine the context for Song2Vector (S2V) embedding. (*D*) The construction of S2V-based distances between songs, people, which represent the centroid of listened song vectors for each listener, cities, which represent the centroid of listening people there, and countries, which represent the centroid of listening within cities around the world. (*E*) S2V cosine similarity between country-level taste vectors: Australia (AU) and the United Kingdom (UK) are closest (0.92), while France (FR) and Brazil (BR) are furthest (0.19).

To systematically evaluate the impact of geographic and routine changes on listening habits, we obtained extensive listener data from Deezer, the seventh largest music streaming service globally (18 million users, including 10 million paying users) whose strategy has focused on identifying and streaming regional music in addition to global hits. The raw data from Deezer consist of complete listening histories from January 2018 to December 2020 for 44,794 randomly selected users across nine countries—France, Germany, the UK, Brazil, Australia, Russia, South Africa, Morocco, and Mexico. Our sample includes approximately 10,000 users from each of the first four countries, whose subscriber bases are relatively large, and about 1,000 users from each of the other five countries with smaller subscriber accounts. Female users account for 27.3% of the total. The data include over 596 million streams of 4,276,197 unique songs. Fig. 1*A* shows our focal countries, the number of users sampled in each country, and the number of streams from those users during our observation period. Fig. 1*B* provides an excerpt of the data collected for each streamed song.

Detailed streaming data allow us to create taste profiles that represent individuals, cities, and countries over time. We created these profiles using a neural network model previously developed to capture similar word meanings in the form of proximate numeric vectors to represent similar songs based on their relative proximity across playlists—the sequences of songs to which users listen. Inspired by collaborative filtering algorithms that exploit individuals’ consumption sequence information (30–34), we apply the Word2Vec algorithm, designed to identify word meaning from context (30, 35, 36), in order to identify song similarity from context. Specifically, we draw on users’ listening sessions containing multiple songs and their sequence to produce embeddings for songs in a latent space. We call this song-embedding Song2Vec (henceforth S2V). The overall process of constructing S2V is summarized in *Materials and Methods*, along with a demonstration of its high correlation (∼0.6) with a musical distance metric based on sonic features alone.

With S2V, we measure qualitative similarity or dissimilarity between songs—akin to the way Word2Vec measures semantic distance between words. As embeddings can be aggregated at different levels of analysis (e.g., user, city, country), S2V is also capable of capturing cross-level relations (e.g., song–user, song–city, user–city, user–country, etc.) and within-level relations (e.g., user–user, city–city, and country–country relations). We define each user’s taste vector as the multivariate mean or centroid of S2V vectors consumed by a given user. In a similar manner, we define a city’s taste vector as the mean of user taste vectors for users living in that city. Finally, we define a country’s taste vector as the mean of city taste vectors for all cities located in that country. Fig. 1 *C* and *D* illustrates the process we use to create them.

In our analyses, we distinguish taste distance from geographical distance. We measure geographical distance between any two cities using the Haversine formula, which determines great-circle distances from longitudes and latitudes, whereas we measure taste distance via the cosine distance between two playlist vectors. Fig. 1*A* shows the geographical and taste distances between London, Rio de Janeiro, and Sydney. Although London is physically closer to Rio de Janeiro than to Sydney, it is much closer in taste to Sydney. Similarly, we can calculate taste similarity between geographies by subtracting the cosine distance between two locations from 1. Fig. 1*E* displays taste similarities between countries.

We are primarily interested in assessing the effect of spatial and routine disruptions on the evolution of individuals’ cultural tastes. For the geospatial movement of each listener, we first capture their home city—the city in which they listened to the most music in a given year. Then, we measure the physical distance between that home city and all other cities they visit in a given month. This distance is at its lowest (i.e., zero) when a listener stays in their home city for the month, while it is much higher when they make international trips. For the cultural taste change of each listener, we measure how much a listener consumes songs qualitatively different from those they listened to in the past, which we capture by calculating the cosine distance between the current month’s user vector and their vector from the prior 6 mo. Taste distance is lowest (i.e., close to zero) when a user listens to a set of songs on repeat that they regularly listened to over the prior half-year, but it is much higher (i.e., close to one) if they explore entirely new songs from genres, artists, or styles that dramatically differ from their usual listening habits.

## Results

### Travel Distance, Taste Exploration, and Taste Resonance.

**Fig. 2.**
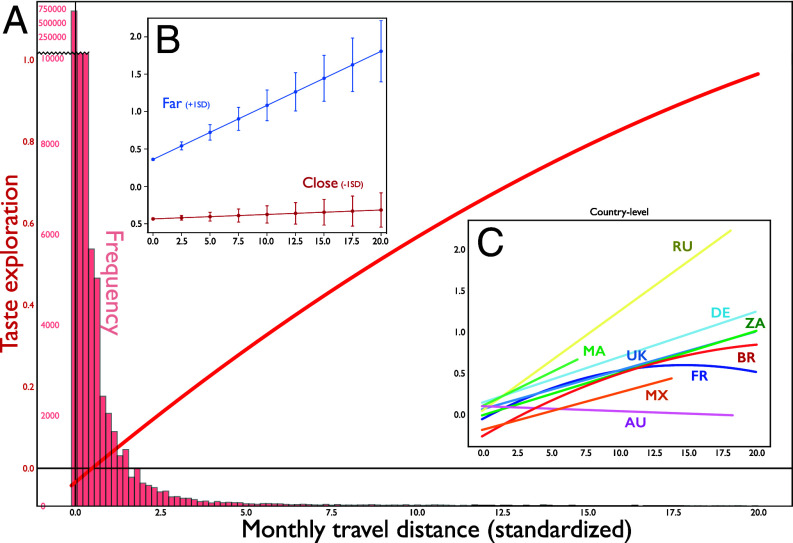
Travel distance and taste exploration. (*A*) Physical distance from listeners’ “home city” has a positive association with their monthly taste exploration, suggesting that the novelty of their listening preferences scales with their geographical exploration. (*B*) This effect positively interacts with their tendency to deviate from global taste. A listener is measured as distant from global taste if her taste vector is one SD beyond the average distance from the global vector in a given month as measured by cosine distance and close if one SD closer. This analysis demonstrates that the positive effect of travel distance on taste exploration is higher among deviants than conformists. (*C*) Same as Fig. 2*A*, but for each country.

Regression analysis of users’ taste evolution at both global and country levels shows that the greater the user’s geospatial movement, the greater the evolution of their taste profile. We found a consistent pattern from 2018 to February 2020, prior to the COVID-19 pandemic’s impact: Individuals who underwent more significant geospatial changes exhibited a higher tendency to engage with cultural products novel to them (bold red line in Fig. 2*A* and *SI Appendix*, Table S1). A similar pattern appears across countries, although the effect in some small-sample countries is not statistically significant. Nevertheless, we see a significantly positive association between geospatial change and taste exploration in France, UK, Germany, and Brazil (*Inset* plot in Fig. 2*C* and *SI Appendix*, Table S6). Routine disruption—here in the form of travel—appears to prompt users to try something new. Robustness checks reported in *Materials and Methods* and *SI Appendix* demonstrate that this association is unlikely driven by users seeking novel experiences who engage in both musical exploration and travel.

We examine the interaction between routine disruption and taste deviation from the global taste average to evaluate whether the positive effect of geospatial movement on taste exploration is more salient when a user deviates from popular, mainstream taste. Our regression analysis supports our prediction: The likelihood of taste exploration as a function of geospatial disruption increases with individuals’ deviation from global taste (*SI Appendix*, Models 8–11 in Table S1). Listeners whose taste patterns are already more deviant are more likely to explore widely when traveling than are listeners whose tastes are generally more conforming. We visualize this interaction effect in Fig. 2*B*.

We further investigate the enduring impact or resonance (37) of newly explored tastes in relation to the disruption of routines (see *SI Appendix* for details). Specifically, does the new music explored while traveling remain in listeners’ consumption patterns beyond the disruption itself (i.e., do explored tastes “resonate” with a listener’s future taste)? Similar to findings from the prior analysis, results show a significant positive association between taste resonance and travel distance, with the impact of travel distance diminishing beyond a certain threshold (*SI Appendix*, Models 4–7 in Table S2). Moreover, the positive relationship between routine disruption and taste resonance is reinforced when a listener diverges from mainstream trends (*SI Appendix*, Models 8–11 in Table S2). For illustrative purposes, consider a hypothetical scenario: A London native, typically inclined toward Rock, discovers Opera for the first time. This introduction could occur in two distinct environments—watching a live performance of “Nessun Dorma” on the streets of Soho on her way back home from work in London or experiencing the same performance serendipitously outside the historic opera house, La Fenice in Venice, during her travels in Italy. While the essence of the music remains unchanged in both scenarios, our findings indicate a stronger, more enduring affinity arising from the Venetian exposure, making it more probable for Opera to feature prominently in her subsequent musical repertoire. It highlights that unfamiliar contexts, particularly those deviating from routine, intensify the persistent impact of recently discovered musical tastes on one’s subsequent structure of tastes.

### Taste Distance and Adaptation.

Related to the association between geospatial and taste exploration is the possibility that people assimilate their cultural taste toward the dominant taste in their new/foreign environment. This tendency is likely amplified with greater distance between hometown taste and that of the new environment to which a listener is exposed. We examine this relationship by comparing how much a user’s listening habits move toward the prevailing listening trends of another city as a function of how far that city’s taste is removed from the user’s hometown (see *Materials and Methods* for details).

**Fig. 3.**
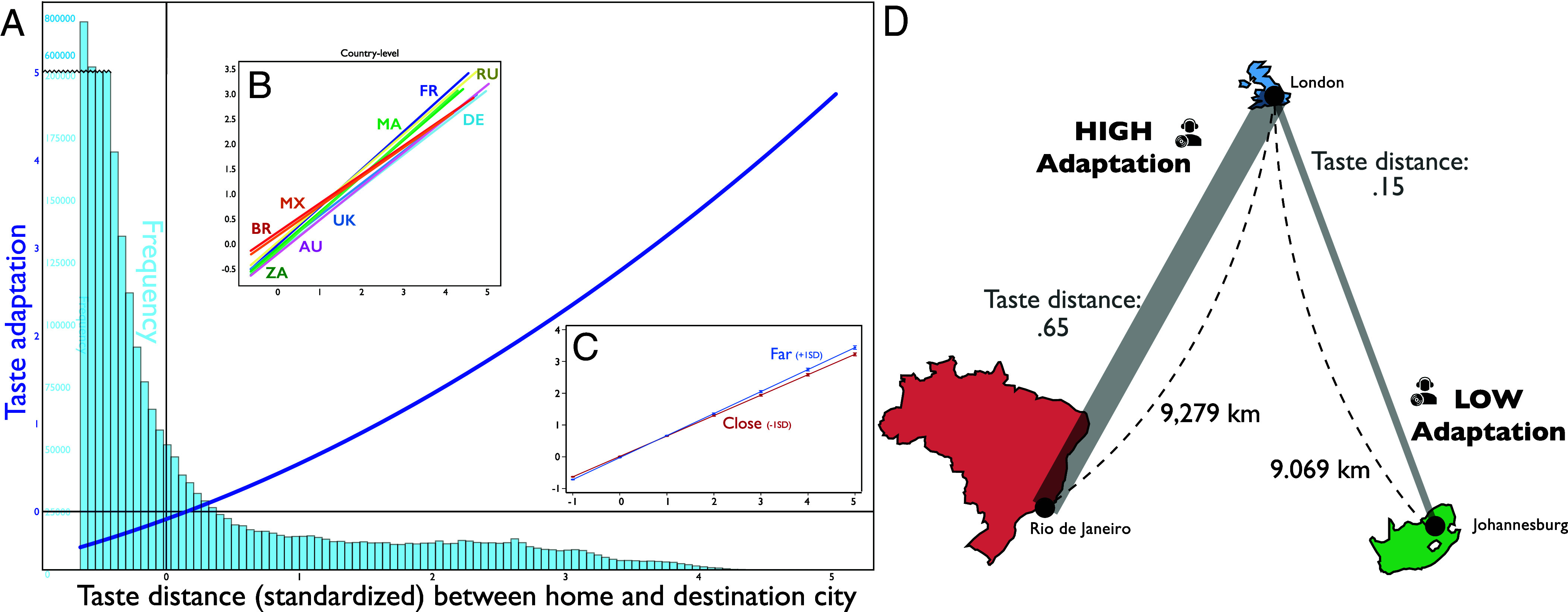
Inter-city taste distance and user taste adaptation. (*A*) A positive association between taste distance from listeners’ “home city” and their destination city suggests that their adaptation to local listening preferences scales with cities’ taste distance, globally, and (*B*) for each country in our sample. (*C*) This effect of taste distance is positively moderated by geospatial distance, where “far” apart cities are one SD greater than average inter-city distances, and “close” cities are one SD smaller than average inter-city distances. (*D*) Example of the average effect in the context of listeners traveling between London and Rio de Janeiro versus Johannesburg.

We find that greater inter-city taste distance between home and host cities is associated with a stronger tendency for the user’s listening to adapt to the new environment (Fig. 3*A* and *B* and *SI Appendix*, Table S3). This is illustrated in an example of a Londoner who travels to both Rio de Janeiro and Johannesburg in Fig. 3*D*. Both cities have comparable geospatial distance from London, but their taste distance from London varies considerably. Our results suggest that this Londoner is more likely to adjust her taste to Rio de Janeiro than Johannesburg because the difference in the tastes between cities is greater when traveling to Rio de Janeiro.

We also examine the interaction between taste distance and geographical distance to evaluate whether the positive effect of taste distance on adaptation is more salient when a user spatially travels further from home. Our regression analysis supports our expectation that the linkage between taste adaptation and taste distance increases with listeners’ geospatial distance from their hometowns (*SI Appendix*, Models 8–11 in Table S3). We visualize this interaction effect in Fig. 3*C*.

### Lockdown and Taste Exploration.

In a typical, pre-COVID-19 setting, the disruptions we examine arise from travel: The farther and longer individuals deviate from home, immersing themselves in unfamiliar settings, the more significant the disruption to their routines. This pattern fits the traditional narrative of how stepping out of comfort zones in the context of “unsettled lives” leads to greater exploration along other cultural dimensions (20). Nevertheless, during the massively disruptive COVID-19 pandemic, confinement replaced commuting, with most individuals bound to the confines of their homes. Normal activities such as regular gym visits, weekly religious gatherings, and weekend socializing were abruptly replaced by the new “normal”: working from home, constant cohabitation with family or pets, and limited social interaction. Under these “unsettled times,” disruption stemmed not from unusually moving away but from unusually remaining at home. Qualitatively different from travel, the effects of COVID-19 confinement were no less disruptive to everyday routines. Fundamentally, both types of disruptions—travel- and confinement-induced—can stimulate exploration, albeit possibly via different mechanisms. The common feature is deviation from routine.

The global pandemic that began in early 2020 provides us with a unique opportunity to examine the impact of these “unsettled times” on consumption patterns. Governments took a wide range of measures in response to the COVID-19 outbreak as it flared up or simmered down. One frequent response, although countries varied in the stringency and timing of their intervention, was the imposition of strict nationwide confinements, which were then lifted several weeks later. The variation in application and stringency provides us with an opportunity to examine whether and how lockdowns affected cultural exploration.

**Fig. 4.**
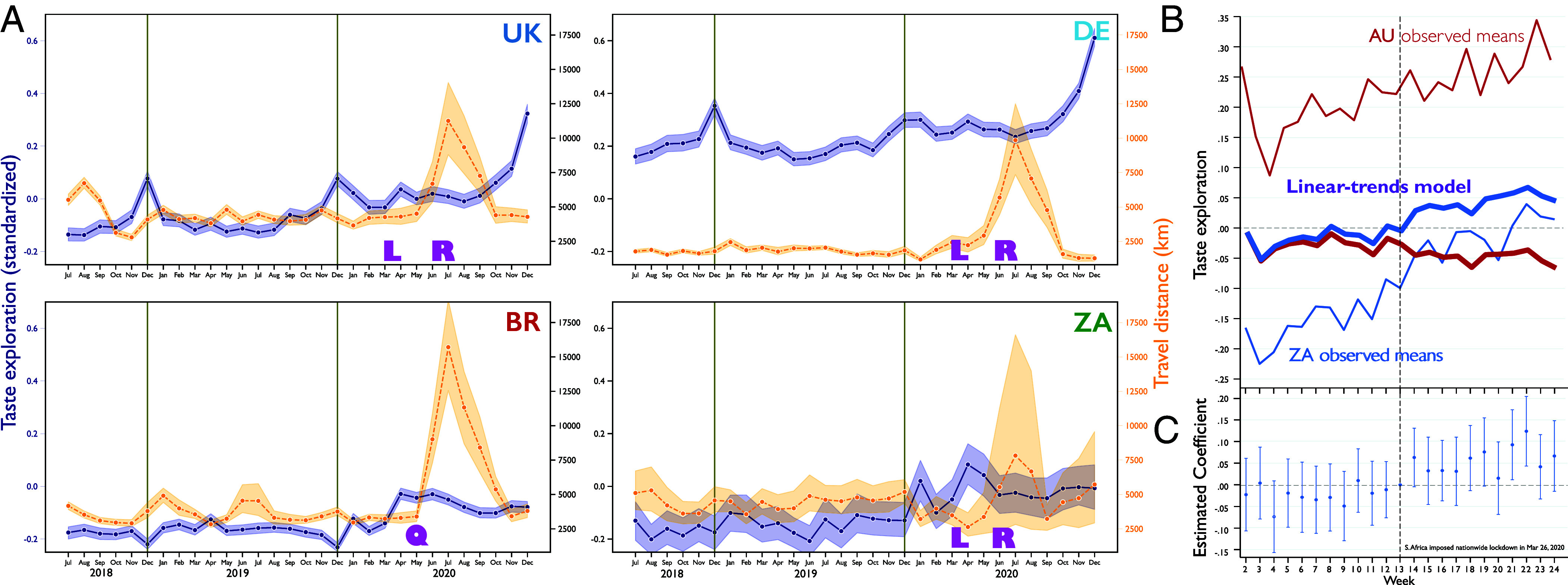
Travel distance, taste exploration, and COVID-19 lockdown. (*A*) Monthly taste exploration and travel distance surrounding COVID-19 lockdown (L), reopening (R), and regional quarantine (Q) events for the United Kingdom (UK), Germany (DE), Brazil (BR), and South Africa (ZA). (*B*) Parallel trends prior to South Africa’s nationwide lockdown in taste exploration between South Africa and Australia. While the observed means of Australia and South Africa show parallel trends prior to the South African lockdown, the latter starts to increase post-lockdown. The linear-trends model further confirms the parallel-trends assumption. (*C*) Week-specific treatment effects of lockdown on taste exploration. This visualizes the result of the Granger causality test that augments our differences in differences (DiD) model to include dummies as if treatment had occurred in the past. It fits a generalization of the DiD model and plots the estimated coefficients with 95% CIs. The contrast between pre- and post-treatment periods in coefficients is aligned with the result of the Granger causality test (*F* = 0.86, *P*-value = 0.583) obtained by performing a joint Wald test on the coefficients.

Users’ taste exploration and travel are relatively stable from 2018 to early 2020 (Fig. 4*A*), with taste divergences around the winter holidays, reflecting increased consumption of holiday music typically not listened to the rest of the year, though trends vary by country (*SI Appendix*, Fig. S1). Travel also increases modestly around the winter and summer holidays. Nevertheless, taste exploration and travel decouple as the pandemic broke out in early 2020. Taste exploration spiked during March and April—months during which the first and strictest lockdowns were implemented—reflecting shifts in people’s tastes when day-to-day routines are disrupted. Taste exploration spikes again directly following the May–June period, when many countries reopened following their first lockdown.

This pattern—a lockdown-related drop in travel followed by reopening, each coinciding with increased taste exploration—suggests a connection between pandemic-induced disruption and the evolving novelty of cultural taste (Fig. 4*A* and *SI Appendix*, Fig. S1). We examine this connection in greater detail via difference-in-differences (DiD) and triple difference-in-differences (DDD) analyses (Fig. 4 *B* and *C* and *SI Appendix*, Table S4). DiD analyses yield separate estimates for selection and treatment effects, enabling us to examine effects of treatments like lockdowns over time across treated and comparison groups.

We highlight South Africa and Australia, similarly represented in our data and which share strong taste similarity (Fig. 1*E* and *SI Appendix*, Fig. S2). In response to the initial outbreak of COVID-19 in 2020, however, South Africa introduced strict, swift measures that disrupted routines more dramatically than did Australia (38). The stringency index calculated by the Oxford COVID-19 Government Response Tracker (39) captured the difference between the two countries. Across the month of March 2020, South Africa’s stringency index increased from the second to the 89th percentile, while Australia’s moved only from the 11th to the 31st percentile. South Africa imposed a 21-d national lockdown with the deployment of military force, effective from 26 March, but Australia never went into complete lockdown. The federal government focused on international travel restrictions (38), leaving implementation of major measures to the discretion of state governments. *SI Appendix*, Fig. S3 visualizes the stringency index of the two countries during the first pandemic period.

For our DiD estimation, we calculate differences in taste exploration before and after the imposition of nationwide lockdown for both countries, visualized in Fig. 4*B*, and then calculate the difference between these differences across the two countries (*Materials and Methods*). Our DiD analysis (Fig. 4 *B* and *C* and *SI Appendix*, Model 7 in Table S4) reveals a strong, positive average treatment effect on the treated group (ATET) (β = 0.081, *P*-value < 0.001, two-tailed), suggesting that South African users who underwent drastic routine disruption due to the national lockdown significantly increased their taste exploration during that lockdown compared to the Australian users whose disruption was less severe.

To add robustness to our DiD analysis, we further employ DDD estimation. This approach capitalizes on the unique opportunity afforded by our data and its detailed user location information to exploit heterogeneity in lockdown restrictions among our treatment group. We create two variables, stuck and inverse mobility, to capture severity in lockdown restrictions. Stuck is a binary variable indicating whether a user’s music-listening activity was limited to a single location for a given week. Inverse mobility, a continuous measure, represents a user’s restricted movement, computed by subtracting the number of distinct cities visited within a week from the highest such count in our data. In this manner, both stuck and inverse mobility quantify the treatment’s intensity. We predict that both stuck and inverse mobility should positively influence taste exploration, a prediction supported by our results (*SI Appendix*, Models 8 and 9 in Table S4). This suggests that individuals with stricter confinement or more limited mobility explored more new music, supporting our hypothesis that geosocial routine disruptions can stimulate novel cultural exploration.

## Discussion

Across settings and contexts, musical preferences reflected rather than compensated for life’s surprises. Disrupted routines as a function of travel systematically associated with increases in exploration and cultural curiosity, influenced by the locations to which listeners traveled. Personal explorations did not veer toward the global center of taste, but away from it and toward distinctive regional music. Furthermore, tastes discovered in more unexpected settings left a stronger imprint on listeners’ subsequent musical tastes and choices. Additionally, we found that unconventional disruptions by COVID-19-induced lockdowns are associated with greater musical exploration. Collectively, listeners appear to construct a soundtrack that mirrors the disruptions of their lives rather than one that counterbalances them. This demonstrates the prescience of Anne Swidler’s theory about how “unsettled times” and “unsettled lives” give rise to innovation and openness to persistent cultural change (20).

Moreover, our study suggests that exploring is not a random pursuit. Exploration is an adaptable behavior, shaped by an inherent need to clarify and understand the nuances of one’s environment (27). A catalyst for such exploration might be uncertainty, which ignites a desire to understand and fit in ref. 40. This is evident when learners encounter scenarios that challenge their pre-existing beliefs or present ambiguities. They tend to be drawn toward experiences or knowledge that seems counterintuitive or puzzling (41). Such patterns offer a potential psychological basis for the preferences we observe among our study’s listeners.

Our study and findings have several limitations. Some of the countries in our sample had uneven listener samples, and there were limits to the amount of global diversity in response to the pandemic we could use as the basis for natural experiments. The patterns raise causal identification questions but in some cases involve forced action imposed exogenously (e.g., lockdowns), which are much more likely to influence listening preferences than the converse. It is also worth noting that the nature of being unsettled varies across individuals and their unique circumstances. Our data and metrics do not encompass extremely disruptive personal events, such as divorce or job loss, paving a path for future research in these areas. Overall, our findings link shifts in convention with changing preferences in cultural consumption. They pose novel measurement opportunities that arise from the signals cultural consumption reveals about the lived experiences and inner states of consumers. Finally, they reveal a pathway through which routine disruptions imprint the evolution of personal tastes and unfolding experiences.

## Materials and Methods

### The Deezer Listener Dataset.

Here, we provide details of the Deezer listener dataset and the analyses conducted. The increasing use of music streaming services and recent availability of data on music consumption on streaming platforms have enabled in-depth research on cultural consumption at global (42), within-country (43), and cross-country levels (44). To the best of our knowledge, no previous study has analyzed a large sample of real-time data on the listening behavior of global users over multiple years.^*^

Compared to Spotify, a top market player in music streaming with 180 million paying subscribers, Deezer has a smaller user base, though the service is available in more countries (185 to Spotify’s 180).^†^ There is no significant difference between Deezer and Spotify with respect to the amount of music content available on the platform: Both platforms have massive music libraries with over 90 million tracks. Nevertheless, Deezer is distinctive in several ways. First, Deezer focuses on distinguishing its platform via localization, providing a large selection of exclusive, original foreign-language music, and podcasts as opposed to exclusively global-reach, international content. For example, Deezer offers 32,000 local and international radio stations, while Spotify does not offer traditional radio on their app. Deezer is also more likely to meet music connoisseurs’ high expectations for audio quality. Unlike Spotify, Deezer uses Free Lossless Audio Codec for paying users of the HiFi option, which allows those users to listen to music with CD-like audio quality. Furthermore, Deezer has a distinctive feature that facilitates music discovery. SongCatcher in Deezer identifies any song playing in the physical vicinity of a listener. It helps users quickly catch and save music playing nearby without leaving the app. This feature provides detailed information about the song followed by the option to add it directly to the user’s library. In sum, with these unique features, Deezer may appeal more to music consumers who value localized, high-quality audio, and the discovery of new sounds.

Among Deezer’s distinctive characteristics, the greater localization of content can be seen, in part, via cross-national diversity in the top music charts across different countries on the app (i.e., the degree to which local songs appear on the chart for the most listened to music, relative to global hit songs). Cross-national diversity or localization is low if many of the same songs appear across different countries’ Top 100 charts, but high when more locally unique songs appear on those charts. We collected the daily most popular 100 songs from Deezer and Spotify in the nine countries we sampled for seven days from December 12 to 18 in 2021. Using the Jaccard similarity coefficient that captures common elements shared by two groups, we show the degree to which the same songs appear across different countries’ Top 100 charts on Deezer and compare the cross-country similarity with Spotify’s cross-country similarity (*SI Appendix*, Fig. S2).

Among country similarities, the lowest Jaccard coefficient is found between France and Brazil, meaning that the French listeners’ music tastes collectively differ from Brazilian tastes more than any other pairing among the nine sampled countries. The three English-speaking countries—the United Kingdom, Australia, and South Africa—share high similarities, likely associated with the linguistic influence that allows for the easy diffusion of foreign music and the emergence of similar taste clusters. This pattern is also seen in the high similarity between Moroccan and French taste. Approximately one-third of Moroccans speak French, and our data reveal that many Moroccan listeners tend to stay in France either for a short (a few months) or long (more than a year) term over the course of our sample. Furthermore, the lower average Jaccard coefficient in Deezer compared to Spotify means that Deezer listeners are more likely than Spotify users to consume local songs over global hits.

In terms of listener demographics, of the 44,794 users in our data, the largest age group comprises those in their 20s (10,835 users), accounting for 24.2% of the total sampled users. The age–gender distribution of sampled users in each country is illustrated in *SI Appendix*, Fig. S4, with 23.7% women listeners.

A variety of information on users’ listening is automatically sent from the listening device to Deezer’s internal server and stored in a log file. Our primary data are derived from those detailed logs of listening behavior at the individual user level. The data used in this study have been fully anonymized by Deezer, with no capability to re-identify individuals given the unique, anonymized user IDs and other variables provided. Accordingly, this study has been classified as “Not Human Subjects Research” by the University of Chicago’s Institutional Review Board and does not require oversight. The listening log data have nine components: 1) anonymized user identifier, 2) track identifier, 3) origin of the listen (e.g., whether playing a song from users’ own collection, through an editorial playlist, or in-app recommendation algorithm), 4) listening date, 5) listening time stamp, 6) duration of the listen, 7) skip identifier (i.e., whether the user clicked ‘next’ before the track ended), 8) platform used (e.g., Android, iOS, web, etc.), and 9) geographical location of the listener at the city level. We use all these elements except platform information (8) to create our central variables.

### Song-Embeddings (S2V).

#### Construction of S2V embeddings.

The data consist of 63.3 million listening sessions of 496 million songs streamed by our sampled users after dropping short-listened streams (i.e., shorter than 1 min). In defining a listening session, we assume that a current listening session, in which a user consecutively listens to multiple songs in a row, ends if there is a break longer than 5 min between the time when the last stream ends and the time when the next stream starts. Session demarcation is important because each session may entail a unique context. For example, a user who jogs along the river in the morning listening to uptempo dance music may play downtempo folk music on her way to work after a considerable session break, such as showering or eating breakfast. As such, our 5-min threshold takes into consideration the possibility that a user’s listening context may change even after a short pause with respect to mood, emotion, situation, etc.

Once we identified the listening sessions, we created our S2V model. Hyper-parameters of the model are set as follows in the Gensim implementation of Word2Vec (45): vector size = 300, minimum count of songs = 2, iteration = 100, skip gram = FALSE, negative sample size = 20, and context window = 3. In other words, the dimensionality of the feature vectors is 300. The maximum distance between the “target” song and its neighboring “context” songs is set to 3, meaning that we train our model such that the vector of the target song is influenced by its three preceding and three following songs in the playlist. Songs must appear at least twice in our data to be included in the training sample. The CBOW (continuous bag of words) model is used instead of the skip-gram model as the training algorithm because even though the number of songs and streams is large in total, the diversity of songs played beside one another means that we require an algorithm robust to data sparsity (46). In addition, 20 negative sample songs (i.e., “noise songs” never played within six songs in a playlist together) are drawn to optimize the quality of the resulting vectors. Even though negative sampling induces statistical bias making it inappropriate for inference, it works well in practice for prediction in word embeddings (47), question answering (48), and many other applications, outperforming the comparable but unbiased contrastive learning approach by co-minimizing bias and variance (47). The result of this operation, run for all streamed songs across our entire sample is a 300-dimensional manifold that embeds songs as a function of their proximity within context-specific playlists. We then assign a unique 300-dimensional vector to each song as a function of their position relative to other songs from our data within the global space of contextual listens.

#### S2V embedding validation.

We initially validated the S2V measure by sampling a focal song and searching for its most similar songs based on the cosine similarity between their S2Vs. Then, we evaluated whether the focal song was more analogous to similar songs musically and contextually than randomly selected songs. We repeated this procedure multiple times. We found that song vectors with higher cosine similarity values are more likely to be categorized as the same style or genre as the focal song, verifying the robustness of our song-embeddings (*SI Appendix*, Fig. S5).

To illustrate our S2V’s validity, we visualize four examples, each of which shows a focal song, its five most similar and dissimilar songs as defined by our S2V model (*SI Appendix*, Fig. S5). Using a *t*-distributed stochastic neighbor embedding (t-SNE), a nonlinear dimensionality reduction algorithm (49), we first reduce a 300-dimensional vector space to two dimensions. We picked one of the most popular Pop/Hip-Hop songs in 2018 (God’s Plan by Drake), a long-running dance song released in 1983 (Billie Jean by Michael Jackson), a non-English Pop song by a Brazilian musician (Bum Bum Tam Tam by MC Fioti), and a chart-topping Alternative Rock song (Creep by Radiohead). Focal songs are marked in black, the most similar in blue, and the most dissimilar in red. Across the four examples, a focal song’s most similar songs are predominantly by the same musician or by other contemporaneous musicians in the same genre, indicating that our S2V model captures both acoustic similarity and other time-varying aspects of songs. In addition, using the entire sample, we compare the level of average cosine similarity within artists and the level of average cosine similarity across artists, expecting the former to be higher than the latter (*SI Appendix*, Fig. S5). The former–within-artist similarity–represents how similar a focal artist’s songs are to each other within that artist. The latter–across-artist similarity–reflects how similar a focal artist’s songs are to all the other artists’ songs in the population. The average within-artist similarity is 0.715, while the average cross-artist similarity is 0.491. The paired *t*-test further confirms the statistical significance of the difference between the two similarity scores (*t*-statistic = 285.033; *P*-value = 0.000).

### Analysis of Taste Exploration (Fig. 2 *A*–*C* and *SI Appendix*, Tables S1, S6, and S7).

#### Dependent variable.

##### Monthly taste exploration.

Our first dependent variable, taste exploration, captures the extent to which a cultural consumer explores novel tastes by measuring the distance between a user’s taste vector in the current month and the mean of that user’s taste vectors over the prior six months (i.e., up to the end of the last month). More formally, user *i*’s taste exploration in month *t*, Taste Exploration_*i,t*_, is measured as the following:

$$Taste\;Exploration_{\mathrm{it}}=cos\;dist\left(UV_{\mathrm{it}},\;\frac{\sum_{t-6}^{t-1}UV_{i}}{6}\right),$$

where $UV_{\mathrm{it}}$ is the user vector of listener *i* in month *t*.

#### Independent variable.

##### Monthly geospatial disruption (travel distance).

Our key independent variable, geospatial disruption, captures the geographic distance a listener covers in a given month upon leaving her home city, assuming she used Deezer at least once during her trip. This first requires specifying one’s hometown or city. We infer a home city for each listener by detecting the city where they used Deezer most. We then identify which cities a listener traveled to while away from home. We measure the monthly travel distance by calculating the Haversine distance between a user’s home city and every city traveled to by that listener in a given month. Then, we sum those Haversine distances per month, resulting in a listener’s monthly total travel distance. For all analyses in which travel distance is interpreted or visualized, we standardize it into its *z*-score.

#### Control variables.

##### Monthly algorithmic guided-ness.

Many music streaming services invest heavily in developing algorithms to provide song recommendations (e.g., “Recommended Playlist,” “Made for Jane Doe,” “Based on your recent listening,” etc.) to their users. Typically, those algorithms generate a list of recommended music that satisfies a given user’s taste but has not been discovered by the user. Nevertheless, it remains up to the user to play that playlist. Idiosyncratic behavioral tendencies to select and endure a playlist are likely to influence the extent to which one explores novelty. We, therefore, control for the degree to which a given user plays music based on algorithmic recommendations per month. It is measured as the ratio of the number of a given user’s streams that came from algorithmic recommendations to her total streams per month.

##### Monthly listening count.

We also consider the total number of a user’s stream counts in a given month to control for the potential effect of listening frequency or duration on a user’s tendency to consume novel music.

##### Monthly average song recency.

Some users have temporal preferences in their musical tastes. For example, a preference for newly released music may affect their propensity for taste exploration. We, therefore, control for the average song recency across a user’s monthly listening sessions. We first calculate a listen-age by counting the number of weeks between a song’s release and when a user listens to it. Then, we subtract the listen-age from the maximum song age in our data to create an inverse song age, which we define as song recency. Monthly average song recency is the mean of song recency for all streams by the user in a given month.

##### Distance from global taste.

Taste exploration also likely varies with a user’s tendency to converge toward or diverge from global trends such as the most popular songs in the world for a given month. We address this likelihood by controlling for each user’s taste distance from the global taste vector—the cosine distance between the focal user’s taste vector and the mean taste vector of all other users in our sample.

##### Month-fixed effects.

To control for time-varying trends, we include month-fixed effects in our model.

##### User-fixed effects.

To control for time-invariant individual characteristics, we include user-fixed effects.

The longitudinal trends of the control variables are visualized in *SI Appendix*, Fig. S6.

#### Empirical strategy and results.

For the analysis of taste exploration, simple OLS regression is not appropriate for two reasons. First, the presence of repeated observations for the same users over time violates the assumption of observation independence. Second, the variance of the error terms might be heterogeneous across different cross-sectional units, yielding unmodeled heteroscedasticity. Therefore, we run a two-way fixed-effects linear model using the xtreg command in Stata 18. We also use robust standard errors, clustering on the user to further account for the presence of repeated observations (50, 51). We standardize all of our variables into *z*-scores before running the model in order to make comparisons between their coefficients interpretable. The descriptive statistics of the variables are reported in *SI Appendix*, Table S5.

The analysis includes data ranging from 2018 to February 2020, excluding during- and post-COVID-19 periods. In doing so, we intentionally leave out any potential influence from the COVID-19 era starting in March 2020. This focus allows us to investigate disruptions in the context of ordinary, nonpandemic periods. This ensures that potential confounding effects stemming from the unique lockdown circumstances are not conflated with the routine scenario.

The global coefficient of monthly travel distance with respect to monthly taste exploration is visualized in Fig. 2*A*, whose inset figure shows the local coefficient for each country. Our focal coefficient is the linear term of travel distance, and it remains significantly positive across the models as shown in *SI Appendix*, Table S1. Its quadratic term is negative, suggesting a concave relationship of diminishing increase between travel distance and taste exploration: Given the differences in magnitude between the two terms, we interpret the results as indicating a positive association between geospatial movement and taste exploration.

We also find a positive interaction between travel distance and user’s deviation from global taste. Its effect remains significant even if any single or pair of countries are removed from the data (e.g., Brazil or/and Australia)^‡^ (*SI Appendix*, Models 8–11 in Table S1). This suggests that the association of geospatial movement to taste exploration is positively moderated by a listener’s tendency to move away from the global taste vector. In other words, one’s exploration of novel taste when traveling is amplified in users with more nonconforming listening patterns. We report the results of country-level regression in *SI Appendix*, Tables S6 and S7.

#### Robustness checks.

First, we test the robustness of our results against several different operationalizations of both the dependent and independent variables. For the dependent variable, taste exploration, we first run the same set of analyses after log-transforming it to assess whether our results are driven by any skewness of its distribution. Results remain substantively the same. Moreover, we apply different time windows for defining a listener’s baseline taste to see whether our results are robust to varied definitions of one’s baseline taste structure.^§^ Concretely, we construct listeners’ baseline taste vectors with streams from only the month prior to a focal month, and we measure taste exploration by the cosine distance between last month’s taste, $UV_{i,t-1}$, and the current month’s taste, $UV_{i,t}$. We also consider the cumulative nature of taste formation by computing a cumulative average of a user’s complete collection of past streams, since the beginning of our observation window (January 2018), up until t−1 month. We then measure their taste exploration by the cosine distance between that cumulative past taste, $\frac{\sum_{1}^{t-1}UV_{i}}{t-1}$, and the current month’s taste, $UV_{i,t}$ . In both cases, our main results remain substantively the same. For the independent variable, travel distance, we substitute the current operationalization—summing all travel distances by a listener in a given month—for the average of those travel distances. This does not substantively change our results.

Second, we replicate our findings by constructing an alternative Song2Vec (S2V) metric that is entirely independent of any contextual or sequential information. Inspired by prior research (52, 53), this alternative representation is based on a comprehensive set of audio features sourced from Spotify, linked to each song in our Deezer dataset via International Standard Recording Codes (ISRC). Spotify’s audio features, constituting a total of 13 distinct dimensions, capture a broad spectrum of sonic characteristics that each song possesses. With these audio features, we develop another S2V representation, which we named “Sonic-S2V.” By employing the Sonic-S2V method, we first recalculate our dependent variable, taste exploration. The correlation between the original S2V-based taste exploration and Sonic-S2V-based taste exploration is high (Pearson *r* = 0.60), reinforcing the validity of our S2V measure. Importantly, a significant positive link between geospatial routine disruption (travel distance) and taste exploration is again confirmed by regression analysis with the alternative Sonic-S2V measure (*SI Appendix*, Table S8).

Third, to test the potential for reverse causality, where taste exploration could incite more travel rather than the converse, we run a Granger causality test (54–56). We execute two separate regressions to inspect the relationship between travel distance and taste exploration. In the first regression model (*SI Appendix*, Model GC 1 in Table S9), taste exploration is the dependent variable, and we incorporate two lagged variables—one representing lagged travel distance and the other indicating prior taste exploration (i.e., the lagged dependent variable). The second model (*SI Appendix*, Model GC 2 in Table S9) makes travel distance the dependent variable and similarly includes two lagged variables—the lagged travel distance (the lag of the dependent variable) and lagged taste exploration. We anticipated that the lagged DVs will be significant in both regression models and that prior travel distance will reveal a significant positive effect on current taste exploration. This would align with our existing findings (as shown in *SI Appendix*, Model GC 1). If traveling is triggered by one’s inclination to expand their listening habits, however, lagged taste exploration should influence current travel distance. As indicated in *SI Appendix*, Model GC 2, we do not observe such an effect. Summing up, this Granger causality test suggests that travel distance is a precursor to taste exploration, but not the converse.

Fourth, we test potential variations in the influence of our independent variable across quantiles of the dependent variable, rather than relying solely on a single average effect estimate derived from our main regression. By employing quantile regression, we explore the nuanced effects of routine disruption across the distribution of taste exploration and do so in a manner robust to outliers. The findings highlight two important observations (*SI Appendix*, Fig. S8). The positive association between routine disruption and taste exploration is consistently significant across all quantiles. Moreover, the magnitude of this effect progressively increases across the distribution of taste exploration. This suggests that while routine disruption generally prompts taste exploration, the effect is particularly pronounced for those who expand their listening habits more extensively (i.e., those in the higher quantiles). Hence, individuals who already have a predilection for exploring a wider array of music tend to experience a stronger impact when their routines are disrupted (i.e., when they travel), further amplifying their exploration.

Finally, we examine whether “radical” taste exploration—distinguished from “average” exploration—is similarly influenced by routine disruption. We define radical taste exploration as the extent to which a listener engages with new songs having cosine distances above a certain threshold from their prior taste centroid. As cosine distance spans between 0 and 1, we initially set 0.9 as the threshold for a new song’s distance from a user’s existing taste centroid to indicate “radical” exploration. When calculating each user’s radical taste exploration, we calculate the proportion of radically novel songs to total songs and use that as our dependent variable. Results (*SI Appendix*, Table S10) reveal a significant positive association between routine disruption (travel distance) and radical taste exploration. This pattern remains robust even with a reduced radicality threshold (i.e., a cosine distance above 0.7), as depicted in *SI Appendix*, Table S11.

### Analysis of Taste Adaptation (Fig. 3 *A*–*D* and *SI Appendix*, Tables S3 and S12).

#### Dependent variable.

##### Taste adaptation to city.

We measure how much a focal user’s taste adapts to the taste of a city she visits. Taste adaptation is high if a user’s taste assimilates to the visited city’s prevalent taste. By contrast, adaptation is low (below zero) if a user’s taste moves further from that city’s taste when visited. Nevertheless, some users might already possess tastes similar to a city they visit (e.g., a French listener who heavily listens to surf rock by the Beach Boys visits Gold Coast in Australia famous for surfing, or a German listener who likes Samba visits Rio de Janeiro where the Brazilian genre originated). In these instances, even though a user does not adjust her taste to that city, her city-specific vector will appear very similar to the visited city, and taste adaptation will incorrectly appear high. To prevent this, we take into account the baseline similarity between a user and a city in our computation of taste adaptation. With this approach, taste adaptation can vary between −1 and 1. The former (i.e., taste adaptation close to −1) corresponds to a consumer whose taste was similar to a focal city’s taste before visiting but becomes distant from that city while visiting. By contrast, the latter (i.e., taste adaptation close to 1) reflects a consumer whose taste was very different from a focal city before visiting but becomes more similar to that city while there. More formally, user *i*’s taste adaptation to a city *c* at month *t*, $Taste\;Adaptation_{i,c,t}$, is measured as follows:

$$\begin{array}{c} \begin{array}{cc} {Taste\;Adaptation\;to\;a\;city}_{\mathrm{ict}} & =\;\cos\;\;\text{sim}\left(UV_{\mathrm{ict}},CV_{c}\right)\\ & \;-\cos\;\;\text{sim}\left(UV_{\mathrm{ih}},CV_{c}\right), \end{array} \end{array}$$

where $UV_{\mathrm{ict}}$ is the user vector of listener *i* based on music that the user played while she was in a city *c* at month *t*, $CV_{c}$ is the city taste vector *c*, and $UV_{\mathrm{ih}}$ is the user’s taste vector in home city *h*. Unlike taste exploration in our first analysis that was computed on a monthly basis, this variable is measured at the visit level; that is, a user can have many data points in a given month if he/she visited many different cities.

#### Independent variables.

##### Taste distance between home and city.

Our key independent variable, taste distance between home and city, captures the inter-city taste distance between a focal user’s hometown and the city to which that user is exposed. We measure the cosine distance between the centroid (or mean vector) of a focal user’s home city and that of a non-home city the user visited. We use this cosine distance as a proxy for the difference in musical environment between a visited city and the user’s hometown.

##### Geographical distance to city.

As our first analysis of taste exploration suggests, it is possible that a user’s taste adaptation is also influenced by geospatial change from hometown to destination city. We measure the physical distance from a user’s home city to a city she visits as the Haversine distance between the two.

##### Control variables.

We also include the same set of covariates as in our analysis of taste exploration. They include distance from global taste, song recency, listening count, algorithmic listening, month-fixed effects, and user-fixed effects.

#### Empirical strategy and results.

We implement a two-way fixed-effects linear model using the xtreg command in Stata 18 to estimate the association between taste distance and a user’s taste adaptation. We use robust standard errors clustered on the user, and we standardize all variables before running the model. Descriptive statistics for all variables are reported in *SI Appendix*, Table S5.

In Fig. 3*A*, we visualize the relationship between inter-city taste distance and user taste adaptation, with insets depicting country-level coefficients. Across the models, we see a positive association between the two, suggesting that the more a visited city’s taste environment differs from a user’s hometown environment, the more that user will adjust their listening preferences toward the place visited (*SI Appendix*, Table S3). A similar pattern appears across countries (*SI Appendix*, Table S12). Furthermore, the interaction between taste distance and geospatial distance indicates that the positive relationship between taste distance and adaptation is positively moderated by long-distance travel (*SI Appendix*, Models 8–11 in Table S3 and Fig. 3*C*). These interaction effects are less prominent in the five countries from our sample with fewer users (*SI Appendix*, Models 5–9 in Table S12).

### Analysis of Lockdown Shock: Difference-in-Differences Estimation (Fig. 4 and *SI Appendix*, Table S4).

For our DiD estimation, we examine differences in taste exploration between Australian and South African users before and after the imposition of a nationwide lockdown in South Africa. To test whether a nationwide lockdown changed the taste exploration trajectory of listeners, we estimate:

$$\begin{array}{c} \begin{array}{cc} Y_{\mathrm{it}} & =\beta_{0}+\beta_{1}\;{\text{treated}}_{i}+\beta_{2}\;{\text{post}}_{i}+\beta_{3}\;{\text{treated}}_{i}\times \;{\text{post}}_{t}\\ & \;+\beta_{4}\;{\text{controls}}_{it-1}+u_{i}+v_{t}+\varepsilon_{\mathrm{it}}, \end{array} \end{array}$$

where $Y_{\mathrm{it}}$ is the taste exploration of listener *i* in week *t*, $\;{\text{treated}}_{i}$ equals one if a listener *i* is a South African who resided in South Africa from January to June 2020 and zero if a listener is an Australian who resided in Australia during the same period, and $\;{\text{post}}_{t}$ equals one if the current week follows South Africa’s first nationwide lockdown date (zero for the week before it).^¶^ We add $\;{\text{controls}}_{it-1}$, which include five covariates–algorithmic listening, listening time, song recency, distance from national taste, and travel distance–measured on a weekly basis and lagged 1 wk. We also include both user fixed-effects ($u_{i}$) and week fixed-effects ($v_{t}$). $\varepsilon_{\mathrm{it}}$ is the error term. We cluster standard errors at the individual listener level. The coefficient of interest is $\beta_{3}$, which measures the difference in taste exploration ($Y_{\mathrm{it}}$) between treated users and control users. While this setting is not the most ideal to evaluate treatment effects and we are hesitant to draw strict causal inference, the result may be interpreted as the suggestive average impact of routine disruption on taste exploration. Descriptive statistics for the variables are reported in *SI Appendix*, Table S5.

We eliminate from the analysis not only Australians and South Africans who lived abroad during the observation period but also those who had been overseas just prior to our observation window. This means that if an Australian user was residing outside Australia until February 2020 and then returned during the initial stage of the pandemic, we would exclude that user from our analysis. This is because such a user might exhibit behavioral traits affected not only by their domestic geocultural environment like lockdown but also by other recent locations. These external cultural influences could potentially introduce a confounding factor in our analysis. We aim to isolate the effect of exogenous geosocial change, specifically the influence of nationwide lockdowns, on the taste exploration of our users. By eliminating users with recent foreign residence, we ensure that behavioral changes we observe are driven predominantly by lockdown conditions in their home countries and not by recent exposure to other cultural contexts. In this way, our DiD estimation will be able to capture the true impact of the lockdown on taste exploration, making our findings more accurate and reliable.

As a result, our DiD model contains 11,757 observations for 641 users—434 South African users and 207 Australian users—who physically stayed in their home country for the 23-wk period from the second week of January to the first week of June 2020. South African users are the treated group and Australians the control group. Note that unlike the previous analyses where variables are measured on a monthly basis, here we use weekly time windows to more precisely exploit the timing of the nationwide lockdown in South Africa. Results show that the ATET is positive and significant (*P*-value < 0.001), indicating that South African users, who underwent drastic routine disruption due to the national lockdown, consumed more novel music after the lockdown than did Australian users whose routine disruption was less severe. For robustness checks, we also ran several models without the two fixed-effects and/or other covariates in *SI Appendix*, Table S4. None of these different model specifications (dropping user- and week-level fixed-effects in Model 6, removing the five covariates in Model 3) changes the result.

To validate the result of our DiD estimate, we examine whether the trajectories of taste exploration are parallel for the control and treatment groups prior to implementation of the treatment. We check what is known as the parallel-trends or common-trends assumption, an important assumption of the DiD model. The parallel-trends assumption for our estimate is supported by a visual diagnostic and a statistical test. First, a graphical diagnostic using “estat trendplots” in the xtdidregress postestimation commands from Stata 18 shows the means of the outcome variable over time for both groups as well as the results of the linear-trends model (Fig. 4*B*). The graph appears to indicate that the parallel-trends assumption is satisfied: Prior to the nationwide lockdown in South Africa, the average taste exploration for users in Australia and South Africa followed parallel paths. We also perform a test for the linear pre-treatment trends using “estat ptrends” following xtdidregress. The linear-trends model estimates a coefficient for the differences in linear trends prior to treatment, testing the null hypothesis that linear trends are parallel. If that coefficient is 0, the linear pre-treatment trends are parallel. The result indicates that we do not have evidence to reject the null hypothesis of parallel trends (*F* = 0.50, *P*-value = 0.48). Hence, both the graphical analysis and the statistical test support the parallel-trends assumption.

In addition, we perform a Granger-type causality test to evaluate whether treatment effects are observed prior to the treatment (i.e., nationwide lockdown), using “estat granger” in the postestimation commands of xtdidregress in Stata 18. The null hypothesis here is that the coefficients prior to the treatment are jointly 0, meaning that there are no anticipatory effects (i.e., the effects start prior to treatment). Results indicate that we do not have sufficient evidence to reject the null of anticipation of treatment (*F* = 0.67, *P*-value = 0.77), suggesting that the treatment effects in our DiD model are not observed ahead of March 26, 2020.

#### Robustness checks.

We delve deeper into the factors driving taste exploration during the COVID-19 lockdown by implementing a DDD analysis. Given the diverse influences that lockdown measures could have on an individual’s daily routine, isolating the predominant factor is complex. Nevertheless, our dataset enables us to investigate the variation in the intensity of lockdown restrictions among our treatment group (i.e., the South African users during the initial lockdown in 2020). To facilitate this, we created two variables: stuck, a binary variable, and inverse mobility, a continuous analog to stuck.

The stuck variable is a binary identifier assigned a value of 1 if a user’s music-listening activity was limited to only one location within a week, and 0 otherwise. This variable is employed to understand whether South African users subjected to the nationwide lockdown experienced greater disruption in their routine to the extent they were stuck in only one location or less disruption due to permitted mobility, such as work-related travel. Inverse mobility is a continuous variable calculated by first determining mobility as the sum of unique cities a user visited within a week. This mobility is then subtracted from the maximum mobility value within our data to compute inverse mobility. This variable measures the degree of lockdown impact on an individual’s geosocial activities, thus providing an additional gauge of the intensity of routine disruption.

In our DDD model, we predicted that both stuck and inverse mobility would significantly and positively influence taste exploration. Our DDD model (*SI Appendix*, Models 8–9 in Table S4) confirmed these predictions. Models 8 and 9 incorporate stuck and inverse mobility as a DDD interaction term, respectively. Results indicate that users experiencing stricter confinement—as indicated by a significant positive coefficient of each DDD interaction term–tended to explore more new music, thereby supporting our hypothesis that disruptions in geosocial routine can motivate novelty exploration.

In addition, a potential alternative explanation for our lockdown-related findings might be that heightened exploration of novel tastes by South African users during the lockdown was merely a result of more time spent on the Deezer platform, which could mechanically lead to more exploration. To address this alternative explanation, we provide evidence suggesting that taste exploration during the lockdown was not influenced by the amount of time lockdown-impacted individuals spent on Deezer, nor or the number of songs they played (*SI Appendix*, Table S13 and Fig. S10).

## Supplementary Material

Appendix 01 (PDF)

## Data Availability

The Song2Vec model trained using listening streams, as well as panel data from anonymized individual listeners on all variables used across models. Data have been deposited in Open Science Framework (https://osf.io/fm2rd/) (57, 58).

## Footnotes

^*^Real-time data in our setting refer to the user’s listening log delivered immediately from the user’s device to the application server.

^†^As of 2023.

^‡^For the reported analysis, we drop Brazil because its national taste vector appears relatively far from the other eight countries (Fig. 1*E*). We drop Australia because its travel pattern seems idiosyncratic compared to the other eight countries (*SI Appendix*, Fig. S1). Results remain consistent with these removals.

^§^We visualize the longitudinal trends of taste exploration based on different time windows in *SI Appendix*, Fig. S7. Note that our current approach in measuring taste exploration uses prior 6-mo streams as a source for a user’s past taste vector.

^¶^Note that, unlike in the previous analyses in which variables are measured on a monthly basis, here, we use weekly time windows to more precisely exploit the timing of the nationwide lockdown imposed in South Africa.
