# Supplementary material for: Disrupted routines anticipate musical exploration
Source: Proc Natl Acad Sci U S A. 2024 Feb 1;121(6):e2306549121. doi: 10.1073/pnas.2306549121 (PMC10861857; doi:10.1073/pnas.2306549121)
Supplement: Supplementary file 1 — Appendix 01 (PDF) [file pnas.2306549121.sapp.pdf]

Supporting Information for

Disrupted Routines Anticipate Musical Exploration

Khwan Kim, Noah Askin, and James A. Evans

Corresponding author: James A. Evans (E-mail: jevans@uchicago.edu)

This PDF file includes:

Supporting text
Figs. S1 to S11
Tables S1 to S15
SI References

Supporting Information Text

Analysis of Taste Exploration Based on Sonic-S2V. In addition to our primary analysis of taste exploration, we replicate our findings by constructing an alternative Song2Vec (S2V) metric that is entirely independent of any contextual or sequential information. Inspired by prior research (1, 2), this alternative representation is based on a comprehensive set of audio features sourced from Spotify, linked to each song in our Deezer dataset via International Standard Recording Codes (ISRC). Spotify's audio features, constituting a total of 13 distinct dimensions, capture a broad spectrum of sonic characteristics that each song possesses. This suite of features includes several continuous variables such as 'acousticness', 'danceability', 'energy', 'instrumentalness', 'liveness', 'loudness', 'speechiness', 'valence', and 'tempo'. Each of these captures a unique facet of a song, ranging from the level of acoustic instrumentation present to the track's overall tempo or speed. In addition to these continuous features, there are also categorical attributes like 'mode', 'key', and 'time signature'.

For the 10 continuous variables, we implemented a normalization procedure, ensuring that each of these metrics now falls within a 0 to 1 range, thereby maintaining a unified scale (1). Regarding the categorical variables, we chose to convert each category into a set of dummy variables. Specifically, 'mode', which signifies whether the song is in a major or minor modality, was converted into 'mode 0' and 'mode 1', indicative of minor and major respectively. Similarly, 'key', which states the track's key, was transformed into 11 separate dummy variables, with each one denoting a unique key (e.g., C, C sharp/D flat, etc.). Furthermore, 'time signature', which provides insight into the song's rhythmic structure, was also split into five dummy variables, each one representing a unique time signature like 3/4, 7/4, etc. Following this process, we now have a set of 29 variables—10 normalized continuous features and 19 dummy variables—all of which fall between 0 and 1. With these variables, we developed a new S2V representation for each song by transforming it into a single 29-dimensional vector, which we named 'Sonic-S2V.' The values within each vector correspond to a song's specific ratings across the 29-feature array.

In employing the Sonic-S2V method, we took several steps: First, we recalculated our dependent variable, taste exploration. Second, we looked into the correlation between the original S2V-based taste exploration and Sonic-S2V-based taste exploration. And lastly, we replicated our primary regression analysis using the Sonic-S2V variant of taste exploration. Our findings showed a high degree of correlation (Pearson r = 0.60) between the two versions of taste exploration, reinforcing the validity of our S2V measure. Moreover, the primary results, suggesting a significant positive link between geospatial routine disruption (travel distance) and taste exploration, were largely confirmed even with the alternative Sonic-S2V measure (see **Table S8** in SI). We note that the significance of the quadratic term was slightly reduced across the new models. Despite this, our analysis affords us greater confidence in arguing a substantive relationship between routine disruption and taste exploration.

S2V vs. Jaccard Index Approach. Here, we demonstrate that our choice of Song2Vec (S2V) for measuring taste exploration has several advantages over the Jaccard index, a traditional similarity (or distance) measure. We applied the Jaccard index to create an alternative, much simpler, taste exploration measure that simply accounted for the intersection over union of shared songs (Jaccard index) in periods of routine and disruption. The Jaccard index treats each song categorically, as equally and "infinitely" different from every other song. We first report the pairwise correlation coefficients between the three different taste exploration measures (see Table S14): the one by our original S2V method, our alternative Spotify Sonic-S2V approach, and the Jaccard Index. Although the first two methods exhibit a strong correlation, neither shows a significant correlation with the Jaccard variant (ρ=.01; ρ=-.03), lending further support to our assertion that the Jaccard method is likely too coarse-grained to capture the kind of taste exploration we examine in our study. Regression analysis for our Study 1 yields far lower R-squared values and nonsignificant β coefficients in predicting taste exploration when using the Jaccard approach (see Table S15). The Jaccard's oversimplicity does not take into account the context of musical consumption, while S2V accounts for song similarity as a function of co-consumption, i.e., consumed together by the same user(s), in the same playlist(s), at roughly the same time. In short, S2V does not perform simpler measures (like Jaccard). S2V does, however, correspond to equally or more complicated measures (like Sonic-S2V).

Analysis of Resonance of Explored Taste. To examine the possibility that the impact of travel might linger and continue to influence musical tastes even after individuals have returned to their regular routines, we draw upon the measure of resonance introduced by Barron and colleagues in PNAS (3). In their analysis of the influence of surprising elements in speeches during the French Revolution's inaugural parliament, they presented three parameters: novelty, transience, and resonance. Novelty denotes the extent of surprise in a speech's structure, considering prior speeches, while transience measures the degree to which these unexpected patterns dissipate in the future (i.e., the extent to which subsequent speeches do not maintain that pattern). Resonance is derived by subtracting transience from novelty, indicating the extent to which novelty persists in subsequent speeches.

Our measure of taste exploration mirrors the concept of novelty. To quantify transience in our scenario, we calculate the cosine distance between the average vector representation of a focal user's musical preferences in a given month t and the average vector of her preferences over the subsequent six months (t+1 to t+6). High transience (or significant decay) implies that few aspects of the listener's taste in month t are assimilated into her future taste. Subsequently, we subtract taste transience from taste exploration to get resonance. High resonance (of taste exploration) implies that a listener's foray into novel music significantly deviates from her preferences over the prior six months (i.e., high taste exploration), and that the newly explored music guides future preferences by maintaining its influence over time (i.e., low transience).

Thus, taste exploration refers to the extent to which a listener's engagement with new music diverges from her established taste preferences, while taste resonance evaluates how this new musical foray retains its influence in subsequent listening habits.

While taste exploration revolves around the act of musical discovery, taste resonance focuses on the sustained impact of such a discovery. Inspired by Barron et al. (2018), we provide a visualization that illustrates how we measure taste resonance by using two relevant constructs—taste exploration and taste transience (see Fig. S10).

The results, as presented in **Table S2**, show a significant positive association between taste resonance and travel distance, with the impact of travel diminishing after a certain threshold (Models 4-7, which include a quadratic term for travel distance). Moreover, Models 8-11 indicate that the positive relationship between routine disruption and taste resonance–again how much of the novelty explored in a given time period persists into subsequent time periods–is reinforced when a listener diverges from mainstream trends (as captured by a significant positive coefficient in the interaction term). Collectively, these results suggest that the positive influence of routine disruption on taste exploration proves to be more enduring when the travel-related disruption is more noticeable.

Analysis of Timings of Exploration. We conducted a series of analyses where we manipulated the timing of the dependent variable (taste exploration) in our main regression analysis. Doing so allows us to examine whether people who travel more are more likely to be more inclined, in general, to expand their listening taste.

Specifically, we substituted taste exploration (our DV) at time t with the same variable at times t-3, t-2, t-1, t+1, t+2, and t+3. We included travel distance at each time (i.e., t-3, t-2, . . .) as well as other controls and retained travel distance at time t in each of these models. We postulated that the coefficient on travel distance would be stronger in the model where taste exploration and travel distance coincide. The findings from these analyses are displayed in the **Fig. S9**. The coefficient on travel distance is strongest and significant only when it is aligned with taste exploration at time t. However, travel distance at t loses explanatory power when extended beyond its contemporaneous time t. Said differently, travel distance does not appear to exert influence on taste exploration except when the two are happening at the same time. In essence, this analysis also indicates a strong association between taste exploration and concurrent geospatial movement.

Analysis of Effect of Listening Time on Exploration. Regarding our findings that routine disruption during the COVID-19 lockdown led to increased taste exploration, a potential alternative explanation might be that the heightened exploration of novel tastes by South African users during the lockdown was merely a result of more time spent on the Deezer platform. This, as opposed to our assertion that the disruption increased exploration, could naturally and mechanically lead to more exploration. To address this alternative explanation, we provide evidence suggesting that taste exploration during the lockdown was *not* influenced by the amount of time lockdown-impacted individuals spent on Deezer or the number of songs they played.

First, our models in Study 3 account for users' listening time, defined as the duration a user spends on Deezer in a given week. We incorporated this variable to address potential concerns regarding the relationship between consumption intensity and taste exploration. Insignificant coefficients across our models, as evidenced in **Table S4**, indicate that listening time did not significantly affect taste exploration during the COVID-19 lockdown.

Second, if the premise that more time dedicated to music results in heightened musical exploration were valid, there would first need to be a noticeable increase in either the number of songs played or the overall time spent on Deezer by those affected by the COVID-19 lockdown, specifically South African users after March 26, 2020. Our data contradicts this assertion. The lockdown did not bring about an increase in the overall duration of usage on the streaming platform. **Fig. S11** illustrates the weekly song count and listening duration throughout 2020 for both South Africa (treated) and Australia (control). This comparison reveals negligible differences in listening intensity within each country before and after the inception of the lockdown.

Finally, we employed a straightforward method to ascertain if listening time (or frequency) influenced taste exploration during the lockdown. We interacted the listening time (or frequency) variable with the DiD term (i.e., POST $\times$ TREATED) in a triple-DiD (DDD) analysis. Had listening time or frequency been determinants of taste exploration, we would anticipate a significantly positive coefficient. However, our findings shown in **Table S13** suggest that neither variable significantly impacted taste exploration among the lockdown-affected group (Models 3 and 4). Conversely, the severity of restrictions they encountered did have an influence (Models 1 and 2).

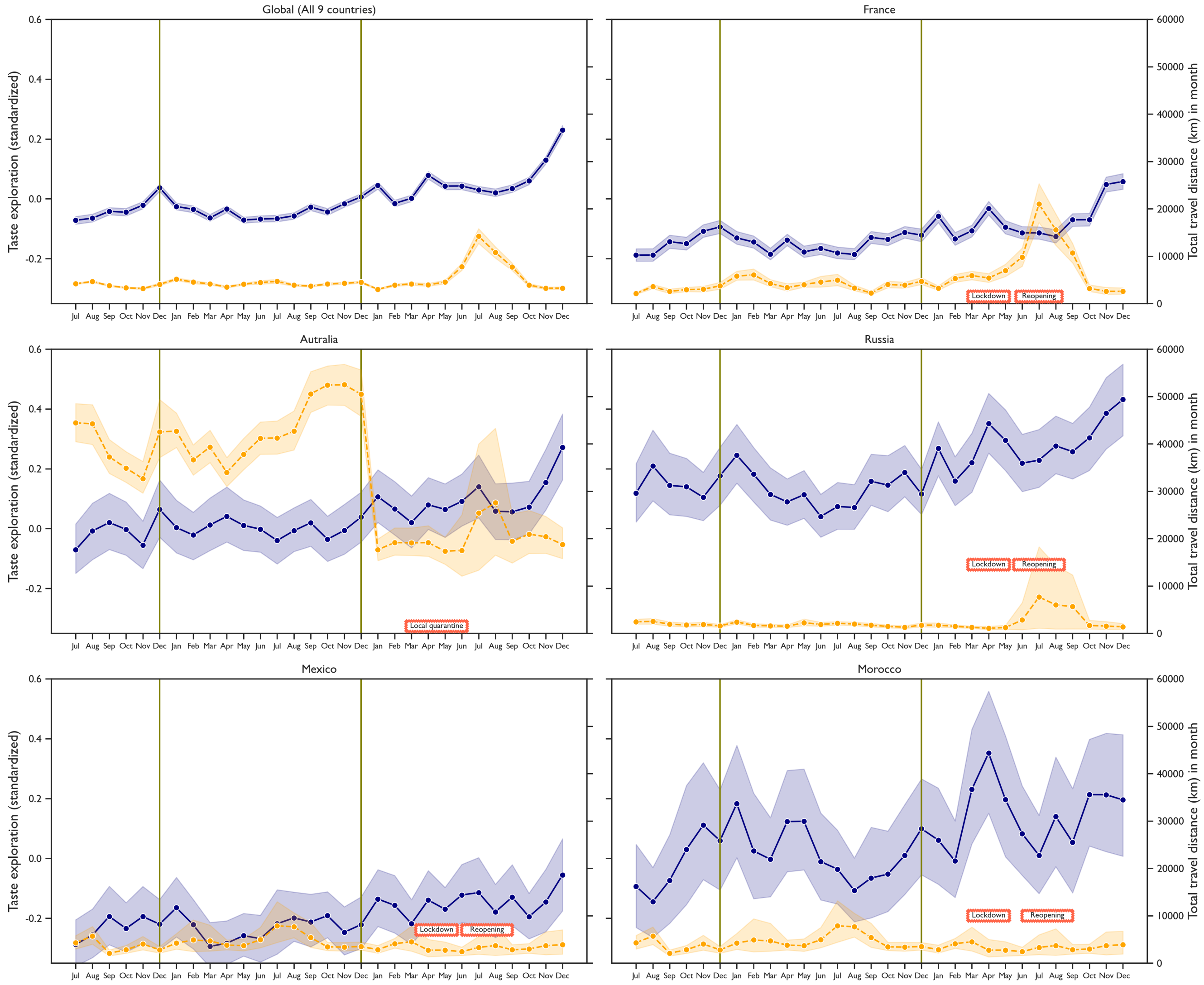

Fig. S1. Taste exploration and travel distance over time. Taste distances are measured by the cosine distance of user vectors between the current month and the prior six months. Travel distances measured by the log of haversine–calculated as km traveled within the month.

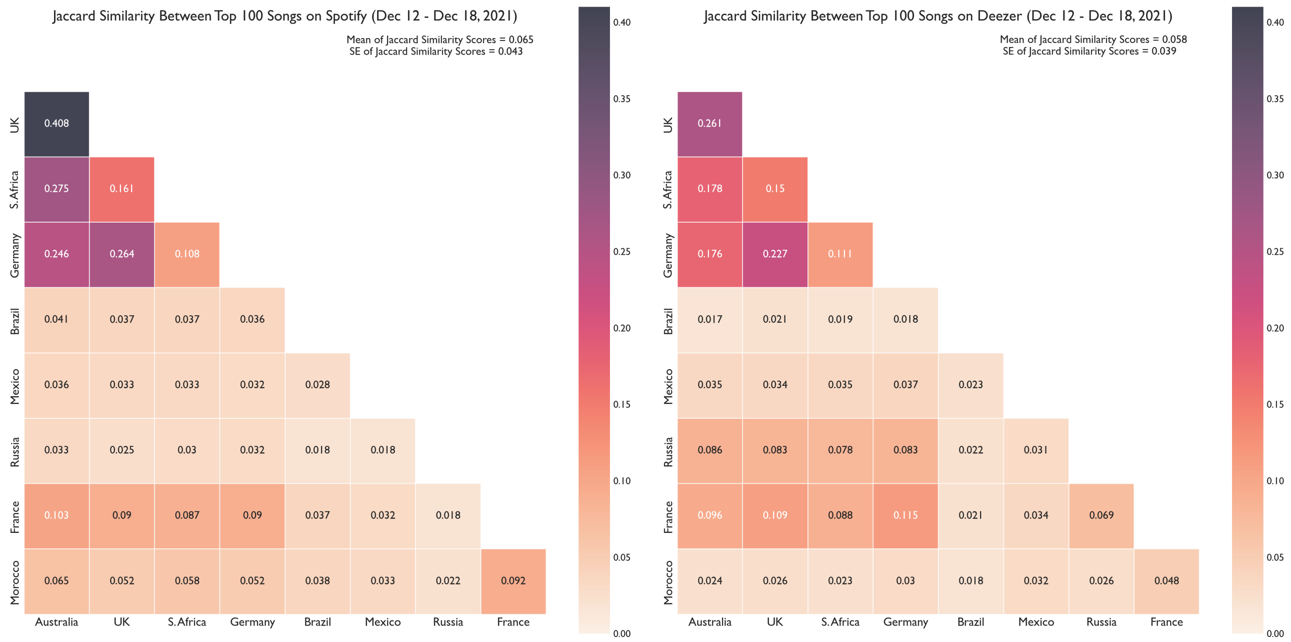

Fig. S2. Jaccard Similarity Between Top 100 Songs on Spotify and Deezer. Cross-country similarity is measured based on what is "popular" in each country. Both Spotify and Deezer release daily charts of the most popular songs by country based on number of streams. We aggregated the top 100 songs from Spotify and Deezer during the same 7-day period and measured inter-country similarity by calculating the Jaccard similarity coefficient.

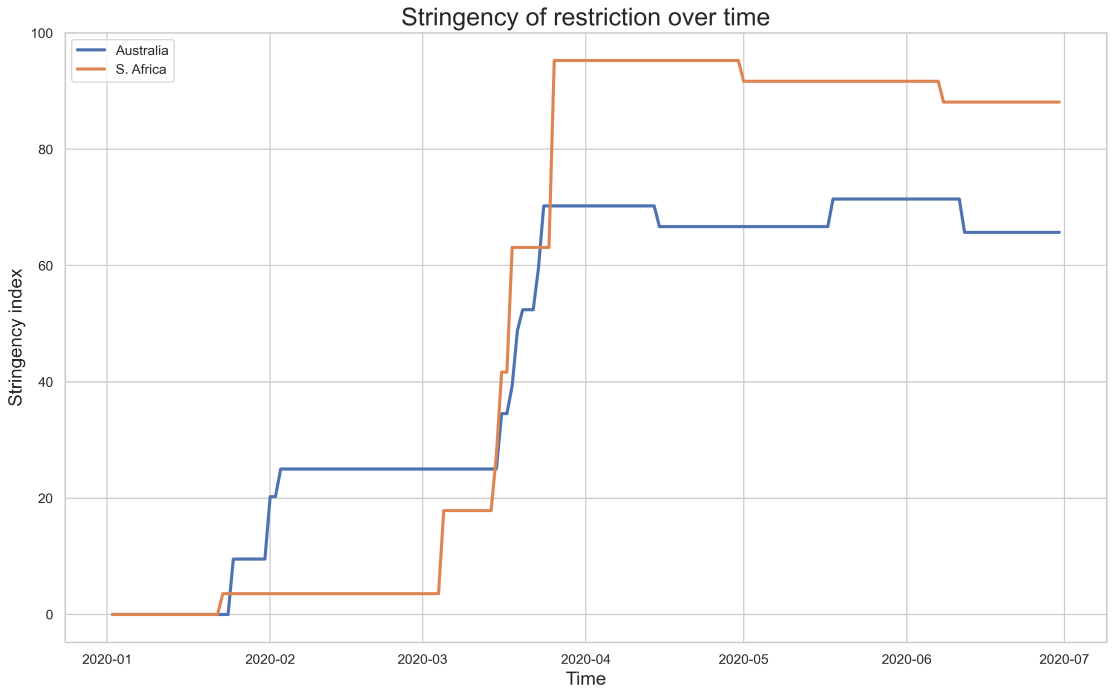

Fig. S3. Stringency of lockdown-related restrictions over the first half year of 2020 in South Africa and Australia. The Oxford Covid-19 Government Response Tracker (OxCGRT) collected systematic information on policy measures that governments implemented to tackle the spread of COVID-19. It tracked various governmental policy responses across different countries from January 1, 2020 to the end of 2022 and quantified them. This includes the stringency index that records the strictness of “lockdown style” policies that primarily restrict people’s behavior ranging from 0 (no restriction) to 100 (maximum restriction). The above figure shows daily scores of the stringency index for South Africa and Australia the first half year of 2020. It highlights a drastic increase in stringency in South Africa compared to Australia in late March 2020, but an earlier (and smaller) stringency bump in Australia when it began to close international borders just before February, likely resulting from its proximity to China.

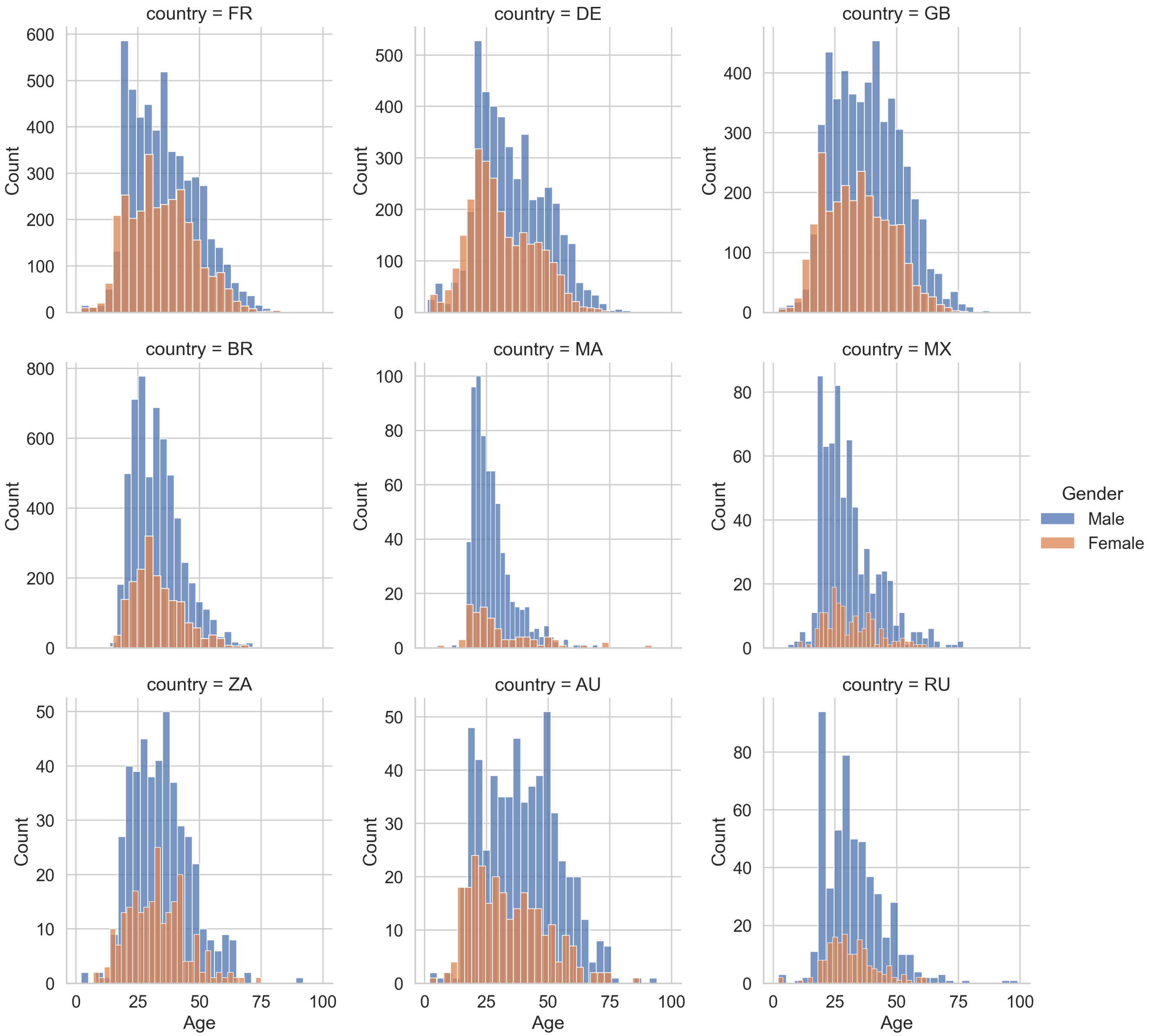

Fig. S4. Distribution of listeners by gender and age. Distribution of the demographic features of our sampled users in each country. Across all 9 countries, male users outnumber female users, and those aged 20-40 account for the largest portion of the sample.

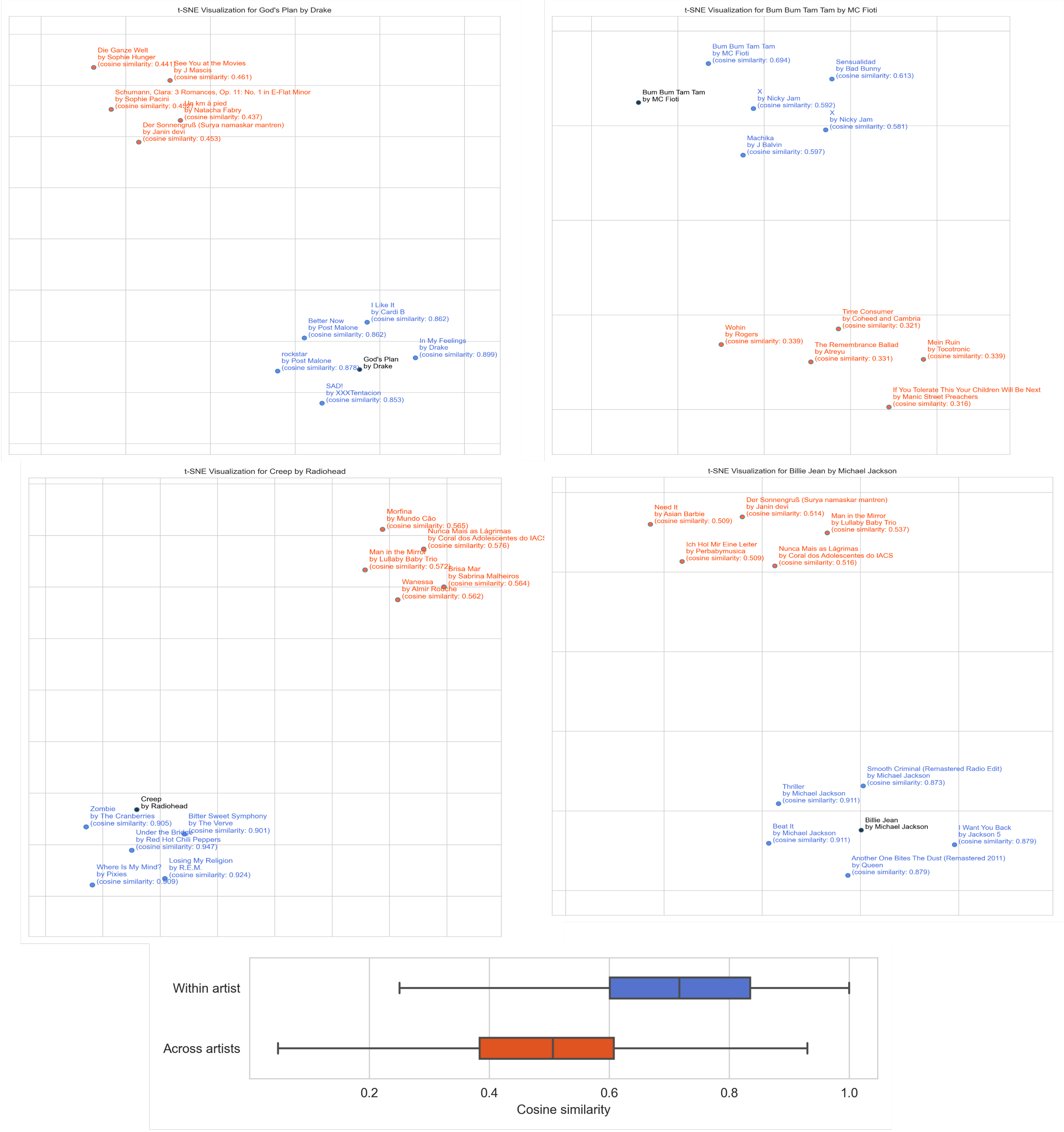

Fig. S5. Validation of Song2Vec (S2V). The 5 most similar and dissimilar songs to a sampled focal song, as predicted by our S2V model, with cosine similarity to that focal song in parentheses. The focal song (in black) in the top left is "God's Plan" by Drake, which debuted at number one on the US Billboard Hot 100 Chart in January 2018. Its most similar songs are closely located (in blue) and are mostly works by other contemporaneous popular musicians in Pop and Hip-Hop. The most dissimilar songs (in red) are remote from Drake's artistic terrain, including an Indie Folk song by a Swiss singer ("Die Ganze Welt" by Sophie Hunger), and one of the Piano Romance Op. 11 that were written in 1839 by a female German pianist, Clara Schumann. Concretely, the average cosine similarity of the five most similar songs to God's Plan is 0.870 whereas that of the five most dissimilar songs is 0.448. Similarly, our model identifies five hit songs by alternative rock bands formed in the 1980-90s as the most similar songs to Radiohead's "Creep" in the top right. At bottom left, a Brazilian rapper MC Floti's 2017 song, "Bum Bum Tam Tam," is positioned closely with other Latin American musicians' hit songs. Note that some of the neighboring songs whose titles are the same as the focal song are different editions of the focal song released in different albums. A mega hit by Michael Jackson, "Billie Jean," has the highest similarity with other famous songs by Michael Jackson or the Jackson 5 of which he was lead member at bottom right. It is also collocated with Queen's "Another One Bites The Dust," the British rock band's unusual disco number. At bottom, using our S2V model, we compare the average cosine similarity of songs within artists and across artists. Specifically, the former is the mean of cosine similarities between songs produced by a focal artist (e.g., similarity between all songs by Michael Jackson). The latter is the cosine similarity between a group of songs by an artist and all songs in the population by all the other artists (e.g., similarity between all Michael Jackson songs and the entire collection of other songs by all the other musicians). The average within-artist similarity is 0.715, while the average cross-artist similarity is 0.491.

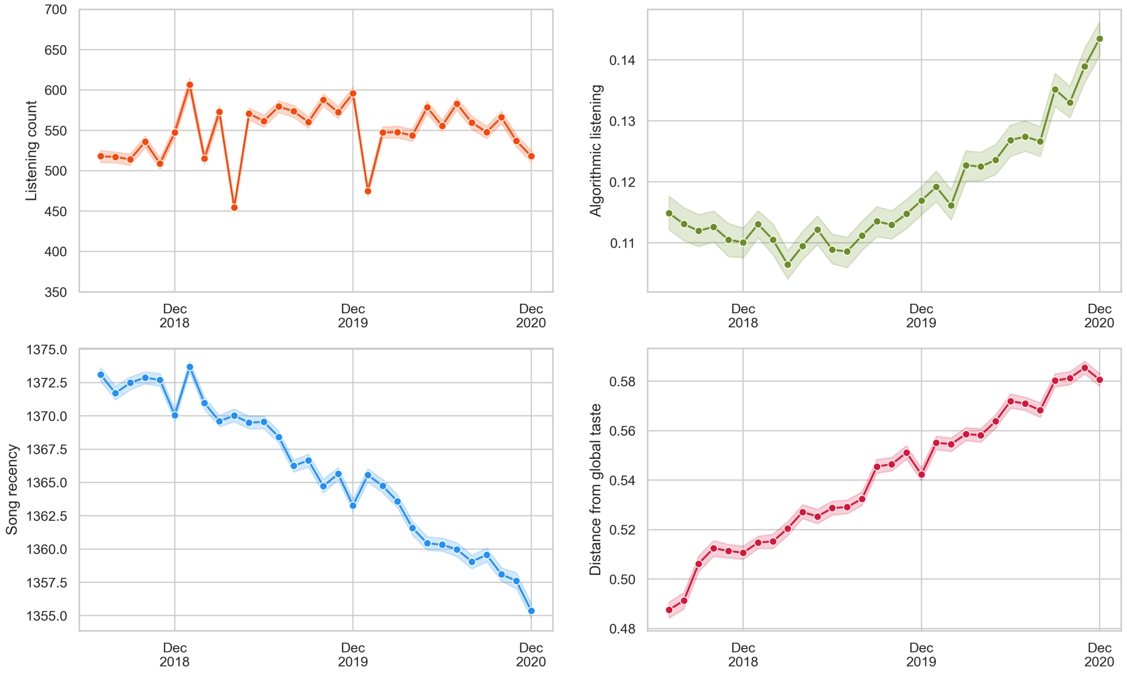

Fig. S6. Longitudinal trends of control variables. Temporal trend of the mean of each of the four control variables included in our analyses across all users in our data. Listening count—defined as the number of total streams longer than 30 seconds each month—hovers between 450 and 600. Algorithmic listening—ratio of algorithm-driven streams to the total streams by user—takes off in 2020 although it stays below 15%. Song recency—the inverse of song age—continues to decline while the most considerable drops occur in two Decembers, presumably driven by classic holiday music. Distance from global taste gradually increases over time although its upward trend also drops in two Decembers, again suggesting a holiday effect.

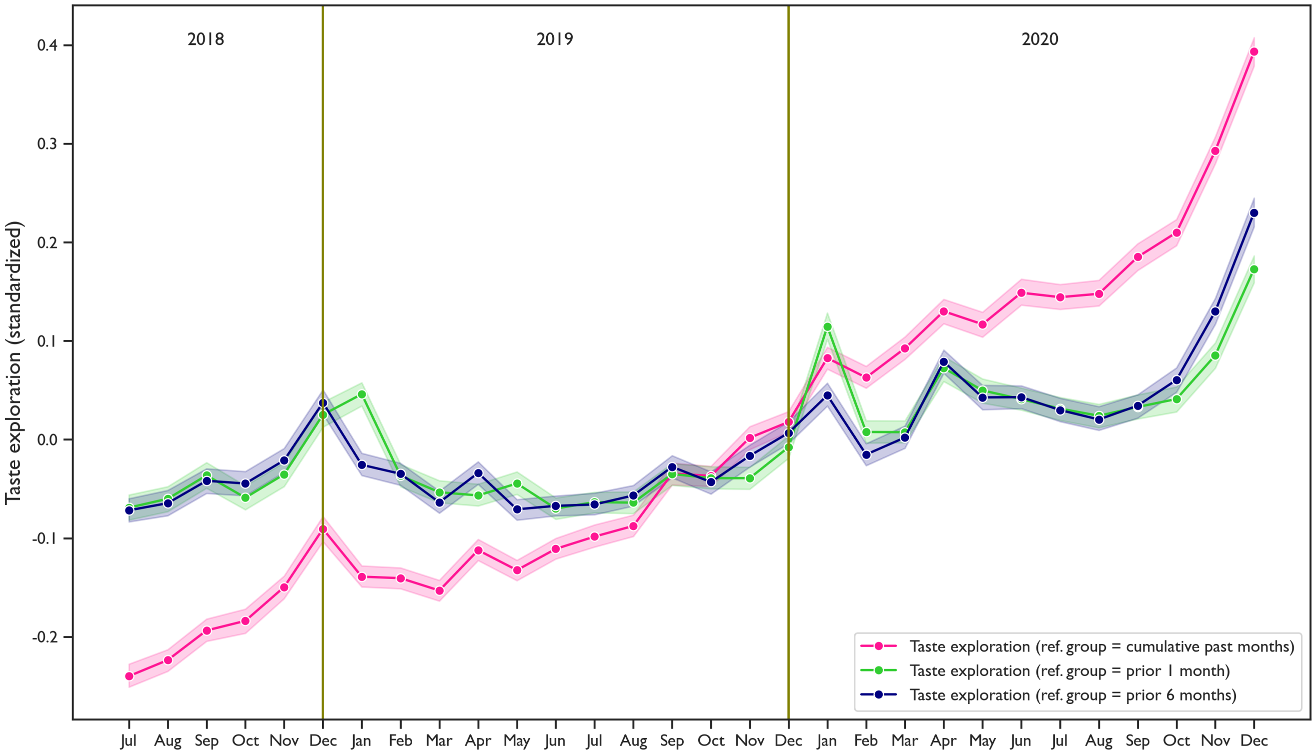

Fig. S7. Longitudinal trend of taste exploration based on different time windows used to calculating baseline taste. We compare taste exploration calculated with respect to the listener's prior month's listening history (green), their prior six months (blue), and their entire history of within-platform listening (pink).

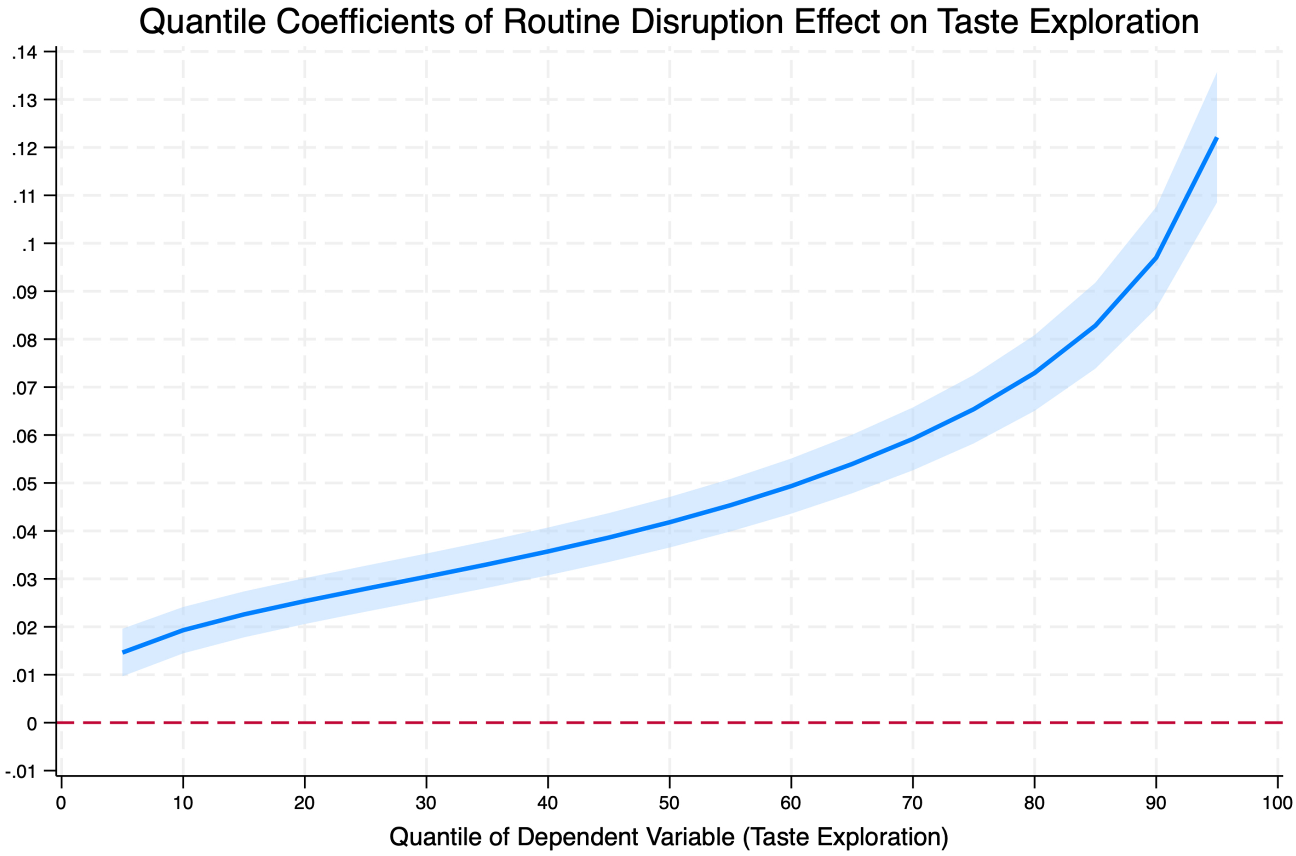

Fig. S8. Results from the quantile regression analysis at different percentiles of the dependent variable, taste exploration (monthly), indicating a progressive increase in the effect of routine disruption across the distribution of taste exploration. The solid line represents the estimated coefficients, and the shaded area around the line indicates the 95% confidence interval. The results highlight the stronger impact of routine disruption on taste exploration at higher quantiles. The coefficients are significantly greater than 0 across all quantiles, suggesting that routine disruptions have a universal effect on taste exploration, but their impact is more pronounced for individuals who are more actively exploring new tastes. Even in the lowest quantiles, routine disruption plays a significant role in pushing listeners out of their comfort zones and prompting them to explore new music, further underscoring the broad and powerful influence of routine disruption on shaping cultural consumption patterns.

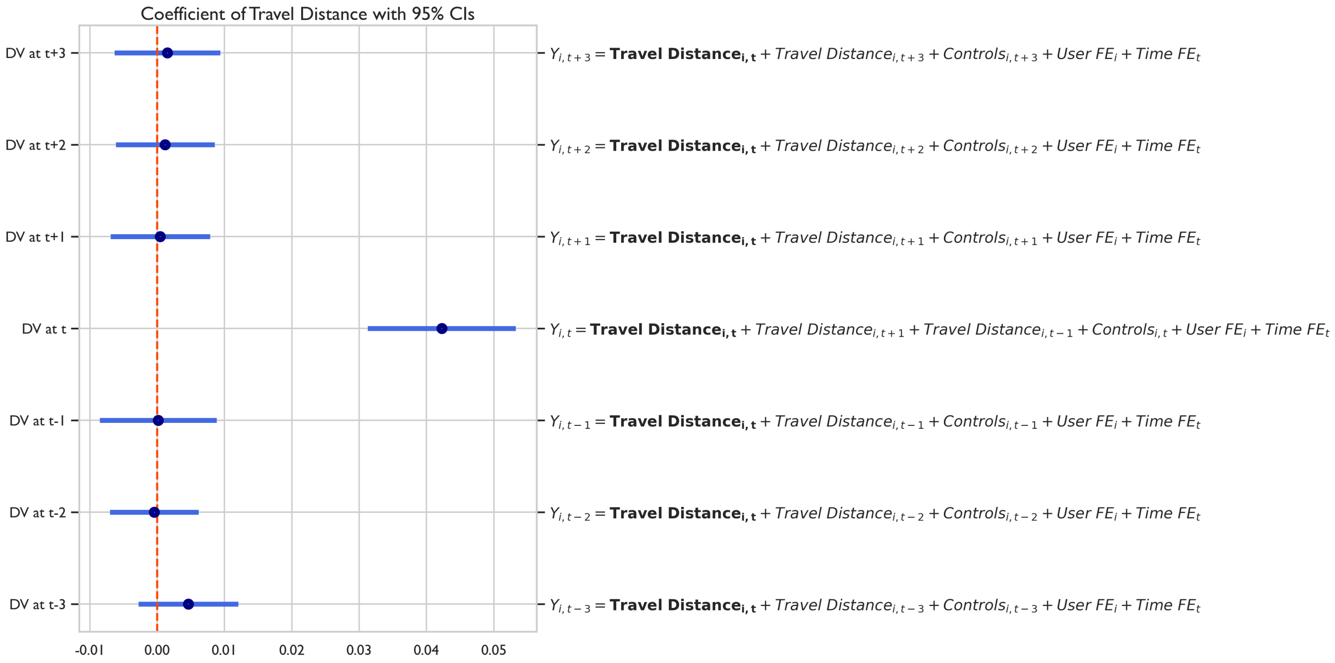

Fig. S9. This plot presents the results from a time-lag analysis examining the relationship between travel distance at t and taste exploration at different time periods (t-3, t-2, t-1, t, t+1, t+2, t+3). Each point represents the coefficient of the travel distance variable (t) from separate regression models, where the dependent variable is taste exploration at a different time period. The strongest and lone statistically significant coefficient is observed at time t (i.e., when the timing of travel distance and taste exploration are aligned). This implies that taste exploration increases in response to the contemporaneous geospatial change.

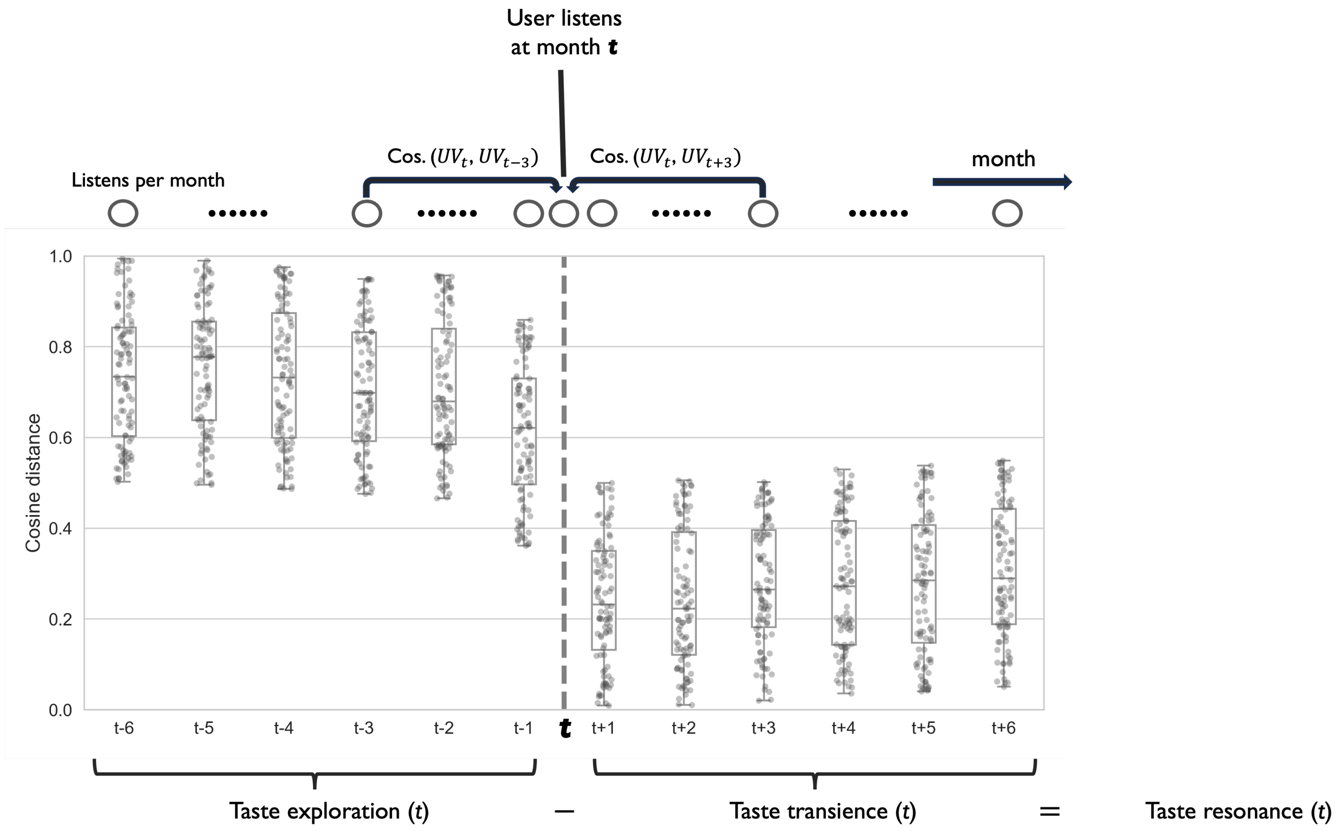

Fig. S10. Taste exploration, taste transience, and taste resonance. To quantify transience in our scenario, in addition to exploration, we calculate the cosine distance between the average vector representation of a focal user's listens in a given month *t* and the average vector of her listens over the subsequent six months (*t+1* to *t+6*). High transience (or significant decay) implies that few aspects of the listener's taste in month *t* are assimilated into her subsequent taste. We subtract taste transience from taste exploration to compute resonance. High resonance (of taste exploration) implies that a listener's foray into novel music significantly deviates from her preferences over the prior six months (i.e., high taste exploration), and that the newly explored music guides future preferences by maintaining its influence over time (i.e., low transience).

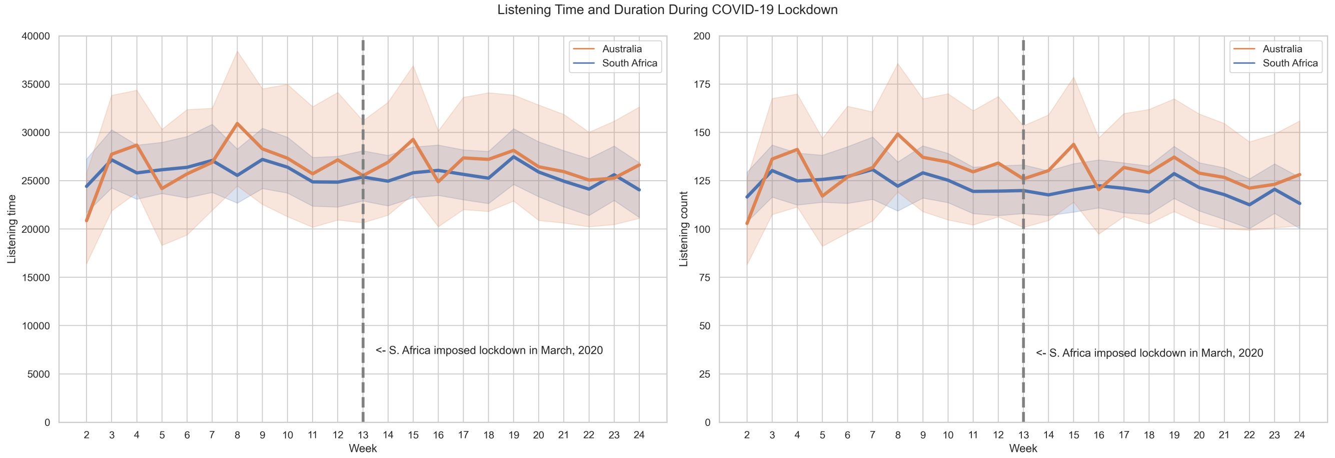

Fig. S11. This plot compares consumption intensity among listeners pre- and post-COVID-19 in South Africa and Australia. The plot on the left shows that there was little difference in listening count among the users, whether in South Africa or Australia, before and after the 26th of March in 2020, when South Africa imposed a nationwide lockdown. Similarly, the plot on the right demonstrates there was no significant change in listening count among the same users. Taken together, this suggests that the lockdown did not lead to an increase in consumption frequency or time on Deezer.

Table S1. Results of regression analysis of taste exploration at the global level. Estimates are from fixed-effects OLS regressions. Cluster-robust standard errors are shown in parentheses; p-values correspond to two-tailed tests. Model 1 includes only the main independent variable—travel distance—and user fixed-effects. Model 2 adds month fixed-effects. Model 3 adds control variables. Models 4-7 add the quadratic term of travel distance. The full sample is used in Model 4, Australians are dropped in Model 5; Brazilians are dropped in Model 6; and both Australians and Brazilians dropped in Model 7 (Brazillians have outlier tastes, and Austrians have outlier travel distances). Instead of the quadratic term for travel distance, Models 8-11 include the interaction term between travel distance and "distance" from global taste.

	DV = Taste exploration in month t										
	Model 1 (No Cov.)	Model 2 (Months)	Model 3 (Linear)	Model 4 (Quadratic)	Model 5 (No AU)	Model 6 (No BR)	Model 7 (No AU&BR)	Model 8 (Interact)	Model 9 (No AU)	Model 10 (No BR)	Model 11 (No AU&BR)
Constant	-.035***	-.079***	-.035***	-.034***	-.034***	.044***	.046***	-.034***	-.035***	.043***	.044***
	(.000)	(.005)	(.005)	(.005)	(.006)	(.006)	(.007)	(.005)	(.006)	(.006)	(.007)
Algorithmic listening			-.049***	-.049***	-.049***	-.050***	-.050***	-.049***	-.049***	-.051***	-.050***
			(.002)	(.002)	(.002)	(.002)	(.002)	(.002)	(.002)	(.002)	(.002)
Listening count			-.230***	-.231***	-.231***	-.223***	-.223***	-.230***	-.230***	-.223***	-.223***
			(.004)	(.004)	(.004)	(.005)	(.005)	(.004)	(.004)	(.005)	(.005)
Song recency			.072***	.071***	.070***	.090***	.088***	.072***	.070***	.090***	.088***
			(.004)	(.004)	(.004)	(.005)	(.005)	(.004)	(.004)	(.005)	(.005)
Distance from global taste			.395***	.395***	.395***	.429***	.429***	.396***	.396***	.429***	.431***
			(.006)	(.006)	(.006)	(.007)	(.007)	(.006)	(.006)	(.007)	(.007)
Travel distance	.034***	.035***	.047***	.070***	.085***	.071***	.091***	.041***	.045***	.042***	.047***
	(.006)	(.006)	(.005)	(.008)	(.009)	(.009)	(.011)	(.005)	(.005)	(.006)	(.007)
Travel distance^2				-.001**	-.002***	-.002**	-.003***				
				(.000)	(.000)	(.001)	(.001)				
Travel distance × Distance from global taste								.033***	.042***	.027***	.039***
								(.007)	(.009)	(.008)	(.011)
User FEs	Yes	Yes	Yes	Yes	Yes	Yes	Yes	Yes	Yes	Yes	Yes
Month FEs	No	Yes	Yes	Yes	Yes	Yes	Yes	Yes	Yes	Yes	Yes
N of obs.	541933	541933	541047	541047	530087	415109	404149	541047	530087	415109	404149
N of users	30384	30384	30339	30339	29725	22922	22308	30339	29725	22922	22308
R^2 between	.000	.000	.192	.193	.192	.230	.229	.192	.191	.229	.228
R^2 overall	.000	.001	.143	.143	.143	.163	.162	.143	.142	.162	.162
R^2 within	.000	.002	.114	.114	.114	.121	.121	.114	.114	.121	.121

Robust standard errors in parentheses are adjusted for clusters in users.

P-values correspond to two-tailed tests.

$^{*}\ p < 0.05$, $^{**}\ p < 0.01$, $^{***}\ p < 0.001$

Table S2. Results of regression analysis of taste resonance. Estimates are from fixed-effects OLS regressions. Cluster-robust standard errors are shown in parentheses; p-values correspond to two-tailed tests. Month dummies are omitted from the table due to space limitation. Model 1 includes only the main independent variable—taste distance to city—and user fixed-effects. Model 2 adds month fixed-effects. Model 3 adds control variables. Models 4-7 add the quadratic term of taste distance to city; the full sample used in Model 4, Australians dropped in Model 5; Brazilians dropped in Model 6; both Australians and Brazilians dropped in Model 7 (see Table S1). Instead of the quadratic term for taste distance to city, Models 8-11 include the interaction term between taste distance to city and geographical distance to city.

	DV = Resonance of explored new taste in month t										
	Model 1 (No Cov.)	Model 2 (Months)	Model 3 (Linear)	Model 4 (Quadratic)	Model 5 (No AU)	Model 6 (No BR)	Model 7 (No AU&BR)	Model 8 (Interact)	Model 9 (No AU)	Model 10 (No BR)	Model 11 (No AU&BR)
Constant	.004***	.007	.015*	.015*	.014	.013	.011	.015*	.013	.013	.011
	(.000)	(.007)	(.007)	(.007)	(.007)	(.008)	(.009)	(.007)	(.007)	(.008)	(.009)
Algorithmic listening			-.016***	-.016***	-.016***	-.016***	-.016***	-.016***	-.016***	-.016***	-.016***
			(.002)	(.002)	(.002)	(.003)	(.003)	(.002)	(.002)	(.003)	(.003)
Listening count			.117***	.117***	.115***	.121***	.119***	.117***	.116***	.122***	.120***
			(.005)	(.005)	(.005)	(.006)	(.007)	(.005)	(.005)	(.006)	(.007)
Song recency			.069***	.069***	.068***	.072***	.071***	.069***	.068***	.072***	.071***
			(.005)	(.005)	(.005)	(.006)	(.006)	(.005)	(.005)	(.006)	(.006)
Distance from global taste			.169***	.169***	.168***	.180***	.179***	.169***	.169***	.180***	.180***
			(.007)	(.007)	(.007)	(.009)	(.009)	(.007)	(.007)	(.009)	(.009)
Travel distance	.024***	.023***	.006	.021*	.026*	.026*	.032*	.003	.005	.007	.011
	(.006)	(.006)	(.006)	(.009)	(.011)	(.010)	(.012)	(.005)	(.006)	(.006)	(.007)
Travel distance^2				-.001*	-.001*	-.001*	-.001				
				(.000)	(.000)	(.001)	(.001)				
Travel distance								.022***	.025***	.019**	.023**
× Distance from global taste								(.006)	(.007)	(.007)	(.008)
User-FEs	Yes	Yes	Yes	Yes	Yes	Yes	Yes	Yes	Yes	Yes	Yes
Month-FEs	Yes	Yes	Yes	Yes	Yes	Yes	Yes	Yes	Yes	Yes	Yes
N of obs.	483737	483737	482964	482964	473173	371107	361316	482964	473173	371107	361316
N of users	30380	30380	30335	30335	29721	22918	22304	30335	29721	22918	22304
R^2 between	.000	.011	.001	.001	.001	.002	.002	.001	.001	.002	.002
R^2 overall	.000	.001	.004	.004	.004	.005	.005	.004	.004	.005	.005
R^2 within	.000	.002	.013	.013	.013	.014	.014	.013	.013	.014	.014

Robust standard errors in parentheses are adjusted for clusters in users.

$^{*}\ p < 0.05$, $^{**}\ p < 0.01$, $^{***}\ p < 0.001$

Table S3. Results from a regression analysis of taste adaptation at the global level. Estimates are from fixed-effects OLS regressions. Cluster-robust standard errors are shown in parentheses; p-values correspond to two-tailed tests. Month dummies are omitted from the table due to space limitation. Model 1 includes only the main independent variable—taste distance to city—and user fixed-effects. Model 2 adds month fixed-effects. Model 3 adds control variables. Models 4-7 add the quadratic term of taste distance to city; the full sample used in Model 4, Australians dropped in Model 5; Brazilians dropped in Model 6; both Australians and Brazilians dropped in Model 7 (see Table S1). Instead of the quadratic term for taste distance to city, Models 8-11 include the interaction term between taste distance to city and geographical distance to city.

	DV = Taste adaptation to city										
	Model 1 (No Cov.)	Model 2 (Months)	Model 3 (Full)	Model 4 (Full)	Model 5 (No AU)	Model 6 (No BR)	Model 7 (No AU&BR)	Model 8 (Full)	Model 9 (No AU)	Model 10 (No BR)	Model 11 (No AU&BR)
Constant	-.000	.079***	-.005	-.086***	-.084***	-.136***	-.134***	-.016***	-.017***	-.072***	-.077***
	(.001)	(.003)	(.003)	(.004)	(.004)	(.004)	(.004)	(.003)	(.003)	(.003)	(.003)
Algorithmic listening			.004***	.005***	.004***	.002*	.002	.004***	.004***	.002	.002
			(.001)	(.001)	(.001)	(.001)	(.001)	(.001)	(.001)	(.001)	(.001)
Listening count			.028***	.028***	.028***	.026***	.026***	.028***	.028***	.026***	.027***
			(.001)	(.001)	(.001)	(.002)	(.002)	(.001)	(.001)	(.002)	(.002)
Song recency			.028***	.025***	.027***	.020***	.022***	.027***	.028***	.021***	.023***
			(.002)	(.002)	(.002)	(.002)	(.002)	(.002)	(.002)	(.002)	(.002)
Distance from global taste			-.222***	-.223***	-.220***	-.239***	-.237***	-.222***	-.219***	-.239***	-.236***
			(.002)	(.002)	(.002)	(.002)	(.002)	(.002)	(.002)	(.002)	(.002)
Geographical distance to city			-.015***	-.012***	-.013***	-.009***	-.007**	-.021***	-.032***	-.020***	-.037***
			(.001)	(.001)	(.002)	(.002)	(.003)	(.002)	(.003)	(.002)	(.004)
Taste distance to city	.664***	.673***	.681***	.550***	.547***	.558***	.555***	.671***	.672***	.677***	.680***
	(.005)	(.005)	(.005)	(.004)	(.004)	(.005)	(.005)	(.005)	(.005)	(.005)	(.005)
Taste distance to city^2				.082***	.085***	.081***	.083***				
				(.004)	(.004)	(.004)	(.004)				
Taste distance to city								.028***	.039***	.034***	.052***
× Geographical distance to city								(.003)	(.004)	(.004)	(.005)
User FEs	Yes	Yes	Yes	Yes	Yes	Yes	Yes	Yes	Yes	Yes	Yes
Month FEs	No	Yes	Yes	Yes	Yes	Yes	Yes	Yes	Yes	Yes	Yes
N of obs.	2420853	2420853	2420853	2420853	2343730	2038385	1961262	2420853	2343730	2038385	1961262
N of users	30186	30186	30186	30186	29572	22846	22232	30186	29572	22846	22232
R^2 between	.451	.448	.645	.652	.651	.707	.705	.645	.643	.707	.703
R^2 overall	.289	.290	.408	.414	.415	.438	.439	.409	.408	.435	.436
R^2 within	.262	.265	.288	.295	.296	.311	.313	.289	.290	.306	.307

Robust standard errors in parentheses are adjusted for clusters in users.

P-values correspond to two-tailed tests.

$^{*}\ p < 0.05$, $^{**}\ p < 0.01$, $^{***}\ p < 0.001$

Table S4. Results of Difference-in-Differences (DiD) and triple DID (DDD) analyses of taste exploration between South Africa and Australia. Estimates are from fixed-effects OLS regressions. Cluster-robust standard errors are shown in parentheses; p-values correspond to two-tailed tests. We deliberately eliminated those users who were living or temporarily staying outside their respective home countries in the recent past period leading up to our observation window. For instance, we excluded a Australian user if she had resided abroad till February 2020 and came back and stay in Australia during the first pandemic period. As a result, our sample includes 641 users, composed of 207 Australians who resided in their home country from January through June 2020 and 434 South Africans who were residing also in their home country during the same timeframe. Models 1 and 2 include only the treatment dummy and only the dummy for post-treatment periods, respectively. Model 3 shows the average treatment effect on the treated (ATET) without considering control variables and fixed-effects. Models 4-7 include control variables. The result of Model 6 intimates a positive ATET with control variables and without fixed-effects. Model 7 shows the positive ATET when considering both control variables and fixed-effects. Model 8 shows the result of our triple Diff-in-Diffs (DDD) analysis that utilizes dummy stuck as a DDD interaction term. Model 9 shows the results of another DDD analysis that uses inverse mobility as a DDD interaction term.

	DV = Taste exploration in week t								
	Model 1	Model 2	Model 3	Model 4	Model 5	Model 6	Model 7	Model 8	Model 9
Constant	-.455***	-.592***	-.465***	-.585***	-.520***	-.504***	-.586***	-.589***	-.620***
	(.027)	(.019)	(.028)	(.007)	(.012)	(.012)	(.007)	(.008)	(.023)
Algorithmic listening				-.008*	-.007*	-.007*	-.008*	-.008*	-.008*
				(.003)	(.003)	(.003)	(.003)	(.003)	(.003)
Listening time				.004	.005	.005	.005	.005	.005
				(.003)	(.003)	(.003)	(.003)	(.003)	(.003)
Song recency				-.102***	-.106***	-.107***	-.102***	-.102***	-.102***
				(.005)	(.005)	(.005)	(.005)	(.005)	(.005)
Distance from nat'l taste				.352***	.358***	.357***	.352***	.352***	.352***
				(.006)	(.006)	(.006)	(.006)	(.006)	(.006)
Travel distance				.022	.020	.016	.018	.019	.028*
				(.013)	(.013)	(.013)	(.012)	(.012)	(.014)
TREATED (S. Africa)	-.162***		-.184***		-.095***	-.117***			
	(.035)		(.036)		(.015)	(.016)			
POST (Mar 26 - May 31, 2020)		.052***	.019		.019***	-.012	-.019	.015	.231***
		(.008)	(.015)		(.004)	(.009)	(.013)	(.016)	(.060)
TREATED × POST			.044*			.041***	.041***	.008	-.203**
			(.017)			(.010)	(.010)	(.015)	(.069)
POST × Stuck								-.042**	
								(.014)	
TREATED × Stuck								-.007	
								(.012)	
POST × TREATED × Stuck								.041*	
								(.016)	
Inverse mobility									.009
									(.006)
POST × Inverse mobility									-.037***
									(.009)
TREATED × Inverse mobility									-.005
									(.007)
POST × TREATED × Inverse mobility									.036***
									(.010)
User FEs	No	No	No	Yes	No	No	Yes	Yes	Yes
Week FEs	No	No	No	Yes	No	No	Yes	Yes	Yes
N of obs.	11757	11757	11757	11757	11757	11757	11757	11757	11757
N of users	641	641	641	641	641	641	641	641	641
R^2 between	.029	.002	.031	.812	.819	.819	.807	.808	.815
R^2 overall	.019	.004	.022	.770	.776	.777	.766	.768	.773
R^2 within	.000	.011	.012	.666	.665	.667	.667	.668	.668

Standard errors in parentheses are adjusted for 641 clusters in users. p-values correspond to two-tailed tests.

Time range of the observations is from the 2nd week of January to the last week of May in 2020.

$^{*}\ p < 0.05$, $^{**}\ p < 0.01$, $^{***}\ p < 0.001$

Table S5. Summary statistics for variables used in regression analyses

Descriptive statistics for variables used in regression of taste *exploration*

Variable	Obs	Mean	Std. Dev.	Min	Max
Taste exploration	541047	-.038	.959	-.871	9.673
Algorithmic listening	541047	-.101	.969	-.824	1.78
Listening count	541047	.077	.873	-2.671	2.937
Song recency	541047	.071	.941	-13.985	2.819
Distance from global taste	541047	-.064	1.006	-2.085	3.784
Travel distance	541047	-.017	.505	-.116	46.593
Travel distance^2	541047	.256	9.455	0	2170.944

Descriptive statistics for variables used in regression of taste *adaptation*

Variable	Obs	Mean	Std. Dev.	Min	Max
Taste adaptation	2420853	-.116	.87	-3.115	4.33
Taste distance	2420853	-.174	.763	-.646	4.9
Taste distance^2	2420853	.613	1.464	0	24.007
Geospatial distance	2420853	-.113	.888	-.451	5.64
Algorithmic listening	2420853	-.122	.843	-.486	4.453
Listening count	2420853	.344	1.141	-.862	19.475
Song recency	2420853	.181	.874	-11.851	2.819
Distance from global taste	2420853	-.115	.984	-2.111	3.784

Descriptive statistics for variables used in DiD models (South Africa, Treated)

Variable	Obs	Mean	Std. Dev.	Min	Max
Taste exploration	8635	-.082	1.022	-4.892	1.758
Algorithmic listening	8635	-.121	.859	-.476	3.387
Listening count	8635	-.098	.785	-.818	9.38
Song recency	8635	.08	.973	-6.888	1.656
Distance from global taste	8635	-.018	.964	-1.553	3.029
Travel distance	8635	-.173	.066	-.187	1.586
TREATED	8635	1	0	1	1
POST	8635	.491	.5	0	1

Descriptive statistics for variables used in DiD models (Australia, Control)

Variable	Obs	Mean	Std. Dev.	Min	Max
Taste exploration	3122	.227	.898	-2.418	1.805
Algorithmic listening	3122	.064	1.085	-.476	3.387
Listening count	3122	-.05	1.015	-.818	8.798
Song recency	3122	-.221	1.04	-5.908	1.646
Distance from global taste	3122	.049	1.092	-1.592	2.96
Travel distance	3122	-.161	.135	-.187	2.128
TREATED	3122	0	0	0	0
POST	3122	.495	.5	0	1

Table S6. Results of regression analysis of taste exploration at the country level (curvilinear relationship). Estimates from fixed-effects OLS regressions. Cluster-robust standard errors are shown in parentheses; p-values correspond to two-tailed tests. Each model corresponds to a separate regression analysis on each of the nine countries. Model 1 (FR) uses the same model specification but only with samples from French users. Model 2 (UK) also uses the same model specification but with British users, and so on. A most salient pattern that emerges across the models is the positive coefficient of travel distance, although the coefficients for the three countries with small sample size are relatively small and statistically not significant. In addition, coefficients for squared travel distance are significantly negative only for France, United the Kingdom, and South Africa. This leads us to argue that it is more appropriate to see the relationship between travel distance and taste exploration as linear with a concave, diminishing effect rather than as an inverted-U.

	DV = Taste exploration in month t								
	Model 1 (FR)	Model 2 (UK)	Model 3 (DE)	Model 4 (BR)	Model 5 (RU)	Model 6 (MA)	Model 7 (AU)	Model 8 (MX)	Model 9 (ZA)
Constant	-.046***	.069***	.150***	-.253***	.059	.111	.101*	-.181***	-.004
	(.010)	(.011)	(.014)	(.010)	(.042)	(.079)	(.041)	(.037)	(.033)
Algorithmic listening	-.048***	-.050***	-.058***	-.040***	-.058***	-.086**	-.051***	-.009	-.046***
	(.003)	(.004)	(.005)	(.003)	(.014)	(.026)	(.015)	(.014)	(.011)
Listening count	-.227***	-.192***	-.233***	-.259***	-.272***	-.276***	-.215***	-.329***	-.222***
	(.007)	(.008)	(.010)	(.006)	(.026)	(.050)	(.027)	(.035)	(.020)
Song recency	.087***	.139***	.057***	.011	.067	.073	.160***	.168***	.118***
	(.008)	(.009)	(.009)	(.008)	(.035)	(.069)	(.032)	(.044)	(.029)
Distance from global taste	.397***	.504***	.417***	.255***	.317***	.526***	.430***	.203***	.479***
	(.011)	(.013)	(.012)	(.011)	(.035)	(.066)	(.044)	(.054)	(.038)
Travel distance	.088***	.047*	.055*	.095***	.120	.081	-.006	.045	.051
	(.014)	(.023)	(.025)	(.025)	(.135)	(.093)	(.011)	(.086)	(.034)
Travel distance^2	-.003**	.014***	-.002	-.002*	.088	-.019	-.000	.006	-.006
	(.001)	(.004)	(.002)	(.001)	(.048)	(.016)	(.001)	(.043)	(.005)
User FEs	Yes	Yes	Yes	Yes	Yes	Yes	Yes	Yes	Yes
Month FEs	Yes	Yes	Yes	Yes	Yes	Yes	Yes	Yes	Yes
N of obs.	120427	112806	139565	125938	10390	2656	10960	5408	12897
N of users	6682	6219	7621	7417	605	155	614	304	722
R^2 between	.238	.238	.191	.142	.177	.148	.286	.252	.158
R^2 overall	.174	.188	.129	.111	.131	.163	.187	.157	.141
R^2 within	.143	.169	.093	.100	.105	.213	.133	.121	.177

Robust standard errors in parentheses are adjusted for clusters in users.

P-values correspond to two-tailed tests.

* $p < 0.05$, ** $p < 0.01$, *** $p < 0.001$

Table S7. Results of regression analysis of taste exploration at the country level (interaction effects). Estimates are from fixed-effects OLS regressions. Cluster-robust standard errors are shown in parentheses; p-values correspond to two-tailed tests. Travel distance squared in Table S6 is replaced by the interaction term between travel distance and distance from global taste. Although not across all countries, three out of four large-sample countries show highly significant coefficients of the interaction term, in addition to South Africa.

	DV = Taste exploration in month t								
	Model 1 (FR)	Model 2 (UK)	Model 3 (DE)	Model 4 (BR)	Model 5 (RU)	Model 6 (MA)	Model 7 (AU)	Model 8 (MX)	Model 9 (ZA)
Constant	-.051***	.072***	.150***	-.253***	.067	.108	.098*	-.180***	-.005
	(.010)	(.011)	(.014)	(.010)	(.041)	(.079)	(.040)	(.037)	(.033)
Algorithmic listening	-.048***	-.050***	-.058***	-.040***	-.058***	-.085**	-.049***	-.010	-.046***
	(.003)	(.004)	(.005)	(.003)	(.014)	(.026)	(.015)	(.014)	(.011)
Listening count	-.226***	-.194***	-.233***	-.258***	-.272***	-.274***	-.215***	-.331***	-.222***
	(.007)	(.008)	(.010)	(.006)	(.026)	(.050)	(.027)	(.035)	(.020)
Song recency	.089***	.139***	.057***	.010	.067	.074	.159***	.167***	.118***
	(.008)	(.009)	(.009)	(.008)	(.035)	(.068)	(.032)	(.044)	(.029)
Distance from global taste	.397***	.505***	.423***	.256***	.315***	.523***	.446***	.206***	.479***
	(.011)	(.013)	(.012)	(.011)	(.036)	(.066)	(.045)	(.053)	(.038)
Travel distance	.037***	.115***	.040*	.036***	.238*	-.030	-.007	.088	.033
	(.007)	(.015)	(.016)	(.009)	(.102)	(.038)	(.009)	(.066)	(.020)
Travel distance	.032**	.071***	.071**	.054***	-.028	-.060	-.023**	.103	-.006
× Distance from global taste	(.012)	(.016)	(.026)	(.014)	(.130)	(.050)	(.008)	(.076)	(.014)
User FEs	Yes	Yes	Yes	Yes	Yes	Yes	Yes	Yes	Yes
Month FEs	Yes	Yes	Yes	Yes	Yes	Yes	Yes	Yes	Yes
N of obs.	120427	112806	139565	125938	10390	2656	10960	5408	12897
N of users	6682	6219	7621	7417	605	155	614	304	722
R^2 between	.236	.238	.191	.142	.177	.151	.291	.250	.157
R^2 overall	.173	.187	.129	.112	.131	.164	.190	.157	.141
R^2 within	.143	.169	.093	.101	.105	.213	.134	.122	.177

Robust standard errors in parentheses are adjusted for clusters in users.
P-values correspond to two-tailed tests.
$^{*}\ p < 0.05$, $^{**}\ p < 0.01$, $^{***}\ p < 0.001$

Table S8. Results of regression analysis of taste exploration using Sonic-S2V (Spotify audio features). Estimates are from fixed-effects OLS regressions. Cluster-robust standard errors are shown in parentheses; p-values correspond to two-tailed tests.

	DV = Taste exploration in month *t* (based on Spotify Sonic-S2V)				
	Model 1 (Linear)	Model 2 (Quadratic)	Model 3 (No AU)	Model 4 (No BR)	Model 5 (No AU&BR)
Constant	.010	.010	.012*	.054***	.057***
	(.006)	(.006)	(.006)	(.007)	(.007)
Algorithmic listening	-.032***	-.032***	-.032***	-.035***	-.034***
	(.002)	(.002)	(.002)	(.003)	(.003)
Listening count	-.321***	-.321***	-.322***	-.312***	-.312***
	(.005)	(.005)	(.005)	(.006)	(.006)
Song recency	.080***	.080***	.079***	.091***	.089***
	(.005)	(.005)	(.005)	(.006)	(.006)
Distance from global taste	.236***	.236***	.238***	.242***	.244***
	(.006)	(.006)	(.006)	(.007)	(.007)
Travel distance	.016***	.029***	.036***	.024*	.033**
	(.005)	(.005)	(.006)	(.010)	(.012)
Travel distance^2		-.001	-.001*	-.001	-.001
		(.000)	(.000)	(.001)	(.001)
User-FEs	Yes	Yes	Yes	Yes	Yes
Month-FEs	Yes	Yes	Yes	Yes	Yes
N of obs.	483618	483618	473815	371529	361726
N of users	30337	30337	29723	22920	22306
R^2 between	.231	.231	.230	.246	.244
R^2 overall	.126	.126	.126	.126	.125
R^2 within	.074	.074	.073	.065	.065

Robust standard errors in parentheses are adjusted for clusters in users.

$^{*}\ p < 0.05$, $^{**}\ p < 0.01$, $^{***}\ p < 0.001$

Table S9. Results of Granger-causality tests. Estimates are from fixed-effects OLS regressions. Cluster-robust standard errors are shown in parentheses; p-values correspond to two-tailed tests. Month dummies are omitted from the table from space limitations. Travel distance squared in Table S5 is replaced by the interaction term between travel distance and distance from global taste. Although not across all countries, three out of four large-sample countries show highly significant coefficients of the interaction term, in addition to South Africa.

	DV=Taste *Exploration* Model GC1	DV=Travel *Distance* Model GC2
Lagged travel distance	.012**	.376***
	(.004)	(.028)
Lagged taste exploration	.139***	.001
	(.003)	(.001)
Algorithmic listening	-.044***	-.004***
	(.002)	(.001)
Listening count	-.190***	.046***
	(.004)	(.003)
Song recency	.059***	.023***
	(.004)	(.003)
Distance from global taste	.389***	.010***
	(.006)	(.002)
Constant	-.040***	-.004
	(.005)	(.002)
User-FEs	Yes	Yes
Month-FEs	Yes	Yes
N of obs.	442835	442835
N of users	30323	30323
R^2 between	.345	.894
R^2 overall	.221	.406
R^2 within	.130	.147

Robust standard errors in parentheses are adjusted for clusters in users.
* $p < 0.05$, ** $p < 0.01$, *** $p < 0.001$

Table S10. Results of regression analysis of "radical" taste exploration (threshold = .9). Estimates are from fixed-effects OLS regressions. Cluster-robust standard errors are shown in parentheses; p-values correspond to two-tailed tests. The threshold for determining radicality of taste exploration is set to 0.9. That is, one's radical taste exploration is defined as the proportion of the number of songs whose cosine distance from her previous taste preference is greater than 0.9 to the number of total songs she consumed in a given month.

	DV = Radical taste exploration ($>$.9) in month t										
	Model 1 (No Cov.)	Model 2 (Months)	Model 3 (Linear)	Model 4 (Quadratic)	Model 5 (No AU)	Model 6 (No BR)	Model 7 (No AU&BR)	Model 8 (Interact)	Model 9 (No AU)	Model 10 (No BR)	Model 11 (No AU&BR)
Constant	-.045***	-.079***	-.038***	-.037***	-.039***	.070***	.070***	-.038***	-.042***	.068***	.066***
	(.000)	(.005)	(.006)	(.006)	(.006)	(.007)	(.007)	(.006)	(.006)	(.007)	(.007)
Algorithmic listening			-.001	-.001	-.000	-.001	-.001	-.001	-.000	-.002	-.001
			(.002)	(.002)	(.002)	(.003)	(.003)	(.002)	(.002)	(.003)	(.003)
Listening count			.046***	.045***	.044***	.062***	.061***	.046***	.046***	.063***	.062***
			(.004)	(.004)	(.004)	(.005)	(.005)	(.004)	(.004)	(.005)	(.005)
Song recency			-.004	-.004	-.005	-.000	-.002	-.004	-.005	.000	-.001
			(.005)	(.005)	(.005)	(.006)	(.006)	(.005)	(.005)	(.006)	(.006)
Distance from global taste			.188***	.188***	.186***	.225***	.224***	.188***	.186***	.225***	.224***
			(.006)	(.006)	(.006)	(.007)	(.007)	(.006)	(.006)	(.007)	(.007)
Travel distance	.073***	.072***	.066***	.113***	.136***	.122***	.154***	.067***	.074***	.067***	.079***
	(.009)	(.009)	(.009)	(.011)	(.013)	(.013)	(.016)	(.009)	(.010)	(.011)	(.013)
Travel distance^2				-.003***	-.003***	-.004***	-.005***				
				(.000)	(.001)	(.001)	(.001)				
Travel distance × Distance from global taste								-.004	-.004	-.012	-.012
								(.008)	(.010)	(.009)	(.012)
User-FEs	Yes	Yes	Yes	Yes	Yes	Yes	Yes	Yes	Yes	Yes	Yes
Month-FEs	Yes	Yes	Yes	Yes	Yes	Yes	Yes	Yes	Yes	Yes	Yes
N of obs.	484413	484413	483633	483633	473830	371539	361736	483633	473830	371539	361736
N of users	30382	30382	30337	30337	29723	22920	22306	30337	29723	22920	22306
R^2 between	.003	.003	.001	.000	.000	.000	.000	.001	.001	.000	.000
R^2 overall	.002	.004	.002	.003	.003	.004	.004	.002	.002	.004	.004
R^2 within	.002	.005	.022	.022	.022	.028	.028	.022	.021	.028	.028

Robust standard errors in parentheses are adjusted for clusters in users.
* $p < 0.05$, ** $p < 0.01$, *** $p < 0.001$

Table S11. Results of regression analysis of "radical" taste exploration (threshold = .7). Estimates are from fixed-effects OLS regressions. Cluster-robust standard errors are shown in parentheses; p-values correspond to two-tailed tests. The threshold for determining radicality of taste exploration is set to 0.7. That is, one's radical taste exploration is defined as the proportion of the number of songs whose cosine distance from her previous taste preference is greater than 0.7 to the number of total songs she consumed in a given month.

	DV = Radical taste exploration ($>$.7) in month t										
	Model 1 (No Cov.)	Model 2 (Months)	Model 3 (Linear)	Model 4 (Quadratic)	Model 5 (No AU)	Model 6 (No BR)	Model 7 (No AU&BR)	Model 8 (Interact)	Model 9 (No AU)	Model 10 (No BR)	Model 11 (No AU&BR)
Constant	-.069***	-.135***	-.073***	-.072***	-.077***	.060***	.058***	-.073***	-.078***	.058***	.055***
	(.000)	(.004)	(.005)	(.005)	(.005)	(.006)	(.006)	(.005)	(.005)	(.006)	(.006)
Algorithmic listening			.001	.001	.001	.001	.002	.001	.001	.001	.001
			(.002)	(.002)	(.002)	(.002)	(.002)	(.002)	(.002)	(.002)	(.002)
Listening count			.037***	.036***	.035***	.047***	.046***	.037***	.036***	.048***	.047***
			(.003)	(.003)	(.003)	(.004)	(.004)	(.003)	(.003)	(.004)	(.004)
Song recency			-.047***	-.047***	-.049***	-.038***	-.040***	-.047***	-.048***	-.038***	-.040***
			(.004)	(.004)	(.004)	(.004)	(.005)	(.004)	(.004)	(.004)	(.005)
Distance from global taste			.236***	.236***	.234***	.277***	.276***	.236***	.235***	.277***	.276***
			(.005)	(.005)	(.005)	(.006)	(.006)	(.005)	(.005)	(.006)	(.006)
Travel distance	.069***	.068***	.064***	.096***	.115***	.103***	.129***	.063***	.070***	.063***	.073***
	(.007)	(.007)	(.007)	(.008)	(.009)	(.009)	(.011)	(.006)	(.007)	(.008)	(.010)
Travel distance^2				-.002***	-.002***	-.003***	-.004***				
				(.000)	(.000)	(.001)	(.001)				
Travel distance								.005	.006	-.003	-.000
× Distance from global taste								(.007)	(.008)	(.008)	(.011)
User-FEs	Yes	Yes	Yes	Yes	Yes	Yes	Yes	Yes	Yes	Yes	Yes
Month-FEs	Yes	Yes	Yes	Yes	Yes	Yes	Yes	Yes	Yes	Yes	Yes
N of obs.	484413	484413	483633	483633	473830	371539	361736	483633	473830	371539	361736
N of users	30382	30382	30337	30337	29723	22920	22306	30337	29723	22920	22306
R^2 between	.002	.001	.020	.021	.020	.034	.034	.020	.020	.033	.033
R^2 overall	.002	.005	.028	.028	.028	.041	.041	.028	.027	.040	.040
R^2 within	.002	.010	.047	.047	.047	.063	.063	.047	.047	.063	.063

Robust standard errors in parentheses are adjusted for clusters in users.

* $p < 0.05$, ** $p < 0.01$, *** $p < 0.001$

Table S12. Results of regression analysis of taste adaptation at the country level. Estimates are from fixed-effects OLS regressions. Cluster-robust standard errors are shown in parentheses; p-values correspond to two-tailed tests. Month dummies are omitted from the table due to space limitation. The positive coefficient of taste distance to city appears highly significant across all models. Its interaction with geospatial distance to city is also significantly positive for the four big-sample countries—France, United Kingdom, Germany, and Brazil.

	DV = Taste adaptation to city								
	Model 1 (FR)	Model 2 (UK)	Model 3 (DE)	Model 4 (BR)	Model 5 (RU)	Model 6 (MA)	Model 7 (AU)	Model 8 (MX)	Model 9 (ZA)
Constant	-.016*	-.080***	-.160***	.236***	.040	-.137**	-.197***	.168***	-.083**
	(.008)	(.005)	(.007)	(.009)	(.032)	(.043)	(.015)	(.025)	(.026)
Algorithmic listening	.002	.005**	-.002	.017***	-.004	.008	.011*	.022	.004
	(.002)	(.002)	(.002)	(.002)	(.011)	(.012)	(.005)	(.011)	(.009)
Listening count	.022***	.032***	.025***	.040***	.041**	.019	.019***	.046***	.026*
	(.004)	(.004)	(.002)	(.004)	(.014)	(.010)	(.005)	(.007)	(.011)
Song recency	.009	-.017***	.046***	.054***	-.036	.059**	-.037***	-.051**	.007
	(.006)	(.004)	(.002)	(.003)	(.018)	(.022)	(.008)	(.017)	(.011)
Distance from global taste	-.223***	-.307***	-.210***	-.109***	-.200***	-.276***	-.306***	-.135***	-.330***
	(.005)	(.004)	(.002)	(.004)	(.015)	(.018)	(.009)	(.015)	(.012)
Geographical distance to city	-.116***	-.015***	-.034**	-.022***	-.133***	-.088*	-.014***	-.030*	-.029**
	(.012)	(.003)	(.012)	(.004)	(.031)	(.040)	(.002)	(.013)	(.009)
Taste distance to city	.755***	.646***	.648***	.576***	.719***	.747***	.676***	.587***	.720***
	(.009)	(.009)	(.007)	(.018)	(.027)	(.033)	(.025)	(.030)	(.034)
Taste distance to city	.081***	.022**	.059***	.012	-.000	.016	-.012**	-.017	-.008
$\times$ Geographical distance to city	(.009)	(.007)	(.011)	(.007)	(.023)	(.031)	(.004)	(.018)	(.022)
User FEs	Yes	Yes	Yes	Yes	Yes	Yes	Yes	Yes	Yes
Month FEs	Yes	Yes	Yes	Yes	Yes	Yes	Yes	Yes	Yes
N of obs.	594476	477351	815125	382468	16078	18978	77123	13945	25309
N of users	6668	6198	7605	7340	584	155	614	304	718
R^2 between	.739	.794	.667	.702	.510	.724	.795	.581	.775
R^2 overall	.462	.466	.406	.395	.443	.434	.432	.353	.486
R^2 within	.365	.263	.289	.155	.389	.304	.293	.278	.334

Robust standard errors in parentheses are adjusted for clusters in users.
P-values correspond to two-tailed tests.
$^{*}\ p < 0.05$, $^{**}\ p < 0.01$, $^{***}\ p < 0.001$

Table S13. Results of triple DiD (DDD) analysis of taste exploration with listening count and time between South Africa and Australia. Estimates are from fixed-effects OLS regressions. Cluster-robust standard errors are shown in parentheses; p-values correspond to two-tailed tests. Models 1 and 2 are identical to Model 8 and Model 9 in Table S4. Model 3 shows the result of our triple Diff-in-Diffs (DDD) analysis that utilizes listening count as a DDD interaction term. Model 9 shows the results of another DDD analysis that uses listening time as a DDD interaction term. The results suggest that neither time-related factor influenced taste exploration among those affected by the lockdown.

**DV = Taste exploration**	Model 1 (Dummy stuck)	Model 2 (Inv. mobility)	Model 3 (Listen. count)	Model 4 (Listen. time)
POST (Mar 26 - May 31, 2020)	.015	.231***	-.020	-.020
	(.016)	(.060)	(.013)	(.013)
POST × TREATED (S. Africa)	.008	-.203**	.042***	.042***
	(.015)	(.069)	(.010)	(.010)
Dummy stuck	.010			
	(.010)			
POST × Dummy stuck	-.042**			
	(.014)			
TREATED × Dummy stuck	-.007			
	(.012)			
POST × TREATED × Dummy stuck	.041*			
	(.016)			
Inverse mobility		.009		
		(.006)		
POST × Inverse mobility		-.037***		
		(.009)		
TREATED × Inverse mobility		-.005		
		(.007)		
POST × TREATED × Inverse mobility		.036***		
		(.010)		
Listening count			-.038	
			(.023)	
POST × Listening count			.001	
			(.007)	
TREATED × Listening count			-.027**	
			(.010)	
POST × TREATED × Listening count			.004	
			(.009)	
POST × Listening time				.002
				(.005)
TREATED × Listening time				-.019*
				(.008)
POST × TREATED × Listening time				.001
				(.007)
User-FEs	Yes	Yes	Yes	Yes
Week-FEs	Yes	Yes	Yes	Yes
N of obs.	11757	11757	11757	11757
N of users	641	641	641	641
R^2 between	.808	.815	.807	.805
R^2 overall	.768	.773	.768	.765
R^2 within	.668	.668	.669	.668

Robust standard errors in parentheses are adjusted for clusters in users.
P-values correspond to two-tailed tests.
$^{*}\ p < 0.05$, $^{**}\ p < 0.01$, $^{***}\ p < 0.001$

Table S14. Correlations between taste exploration measures using different methods. The pairwise correlation coefficients between the three different taste exploration measures: our original S2V method (S2V), the Spotify Sonic-S2V variable (Spotify sonic), and the Jaccard approach (Jaccard). Although the first two methods exhibit a strong correlation, neither shows a significant correlation with the Jaccard variant, lending further support to our assertion that the Jaccard method is likely too coarse-grained to capture the kind of taste exploration we are examining in our study.

**Correlation coefficients**	Taste exploration (S2V)	Taste exploration (Spotify sonic)
Taste exploration (Spotify sonic)	0.596	
Taste exploration (Jaccard)	0.0141	-0.0304

Table S15. Regression analysis of taste exploration using the Jaccard-based measure. The Jaccard dissimilarity variable yields a noticeable decline in R-squared values across the models, and the statistical significance of the primary predictor variable–travel distance–disappears. Some of the control variables unexpectedly flip the sign of their coefficients. This discrepancy further underscores our concerns about the appropriateness of the Jaccard method for our analysis.

**DV = Taste exploration based on Jaccard index**	Model 1 (Linear)	Model 2 (Quadratic)	Model 3 (No AU)	Model 4 (No BR)	Model 5 (No AU&BR)
Constant	-.018***	-.018***	-.016**	.007	.011
	(.005)	(.005)	(.005)	(.006)	(.006)
Algorithmic listening	.014***	.014***	.014***	.016***	.016***
	(.002)	(.002)	(.002)	(.002)	(.002)
Listening count	-.042***	-.041***	-.041***	-.043***	-.043***
	(.006)	(.006)	(.006)	(.007)	(.007)
Song recency	-.044***	-.044***	-.044***	-.035***	-.035***
	(.004)	(.004)	(.005)	(.005)	(.005)
Distance from global taste	-.115***	-.115***	-.114***	-.112***	-.112***
	(.006)	(.006)	(.006)	(.007)	(.008)
Travel distance	.011	.000	.002	.006	.011
	(.006)	(.006)	(.007)	(.006)	(.006)
Travel distance^2		.001	.000	.000	-.000
		(.000)	(.001)	(.000)	(.000)
User-FEs	Yes	Yes	Yes	Yes	Yes
Month-FEs	Yes	Yes	Yes	Yes	Yes
N of obs.	472832	472832	463027	363612	353807
N of users	29653	29653	29039	22432	21818
R^2 between	.021	.021	.021	.016	.015
R^2 overall	.018	.018	.018	.015	.015
R^2 within	.014	.014	.014	.013	.013

Robust standard errors in parentheses are adjusted for clusters in users.
P-values correspond to two-tailed tests.
$^{*}\ p < 0.05$, $^{**}\ p < 0.01$, $^{***}\ p < 0.001$

References

1. N Askin, M Mauskapf, What makes popular culture popular? product features and optimal differentiation in music. *Am. Sociol. Rev.* **82**, 910–944 (2017).
2. JM Berg, One-hit wonders versus hit makers: Sustaining success in creative industries. *Adm. Sci. Q.* **67**, 630–673 (2022).
3. AT Barron, J Huang, RL Spang, S DeDeo, Individuals, institutions, and innovation in the debates of the french revolution. *Proc. Natl. Acad. Sci.* **115**, 4607–4612 (2018).
